# Phylogenetic classification and genetic insights from the complete mitochondrial genome of *Medicago ruthenica*

**DOI:** 10.3389/fpls.2025.1648505

**Published:** 2025-09-05

**Authors:** Yonglei Tian, Zinian Wu, Chunyu Tian, Yanting Yang, Zhiyong Li

**Affiliations:** ^1^ Institute of Grassland Research, Chinese Academy of Agricultural Sciences, Hohhot, China; ^2^ Key Laboratory of Grassland Resources and Utilization of Ministry of Agriculture, Hohhot, China

**Keywords:** *Medicago ruthenica*, mitochondrial genome, gene transfer, RNA editing, phylogenetic analysis

## Abstract

**Introduction:**

*Medicago ruthenica*, an essential wild perennial relative of *Medicago sativa*, is distributed across Eurasia, with good palatability and strong tolerance to drought, cold, and saline-alkali stress. It is exhibits wide adaptability, and its morphology varies greatly under different environmental conditions. Owing to this rich morphological diversity, the inference of its phylogeny has been controversial. Extensive studies have been conducted on the nuclear and chloroplast genomes of *M. ruthenica*; however, because of the complexity of the mitochondrial genome, the *M. ruthenica* mitochondrial genome has not been systematically described.

**Methods:**

In this study, we successfully assembled the *M. ruthenica* mitochondrial genome and investigated various related aspects, including genomic features, RNA editing sites, codon preference, gene transfer events, phylogeny, selective pressure, nucleotide diversity analysis and collinearity analysis.

**Results and discussion:**

The mitogenome of *M. ruthenica* is a circular DNA molecule measuring 354,988 base pairs (bp) in length and has a GC content of 45.13%. The mitochondrial genome of *M. ruthenica* includes 55 genes, with 34 being unique protein-coding genes (PCGs) (excluding three likely pseudogenes), along with three ribosomal RNA genes and 18 transfer RNA genes. The mitogenome is abundant with repetitive sequences; a total of 148 dispersed repeats were identified, including 79 palindromic repeats (53%) and 69 forward repeats (47%), without inverted or complementary repeats. Leucine (Leu) was the most frequently occurring amino acid in PCGs at 10.63%, while tryptophan (Trp) was the least frequent at 1.46%. The codon AUU (Ile) had the highest frequency of use, with the relative synonymous codon usage (RSCU) value of 1.29. *atp8*, *atp4*, *rps4*, *nad4*, *ccmB*, *ccmFc* and *nad9* showed significant positive selection pressure in multiple legumes (Ka/Ks > 1) Additionally, *M. ruthenica*’s phylogenetic relationship was categorized under *Medicago*, supported by mitogenome data from 33 other legume species. Understanding the mitochondrial genome characteristics of *M. ruthenica* is critical for elucidating its genetic background. The results of this study serve as a basis for the genetic breeding of *M. ruthenica*.

## 1 Introduction

*Medicago ruthenica* is a perennial forage legume distributed across Eurasia (Small and Jomphe, 1989). Widely recognized as a premium protein forage, it is valued for its resilience to cold, drought and barren soils, as well as its trampling tolerance. Characterized by large leaves and soft stems, this forage has exceptional nutritional value and palatability. Notably, it contains no saponins and poses no risk of bloating disease in livestock, making it a highly desirable feed source (Choi et al., 2020; Shu et al., 2018). The crown of *M. ruthenica* can reach 1.5 m, covering extensive grassland areas and effectively reducing soil water and heat loss (Wuriya et al., 1994). Its drought tolerance exceeds that of other leguminous forage species (Li et al., 2010). Therefore, *M. ruthenica* plays a vital role in grassland improvement, artificial grassland establishment, and soil-water conservation.

Owing to its rich morphological diversity, inferences regarding its phylogenetic position have remained controversial. When first recorded in 1753 (Linnaeus, 2015), *M. ruthenica* was classified as *Trigonella* because of its morphological characteristics, growth habit, and chromosome number similarities to *Trigonella* spp., and named *Trigonella ruthenica*. It was reclassified as *Medicago ruthenica* in 1841 (Small and Jomphe, 1989), changed to *Pocockia ruthenica* in 1872 as reported in the Wikimedia Species Database (https://species.wikimedia.org/wiki/Trigonella), renamed *Trigonella korshinskii* in 1945 and *Trigonella emodi* in 1955 (Latch, 1976), reclassified as *Melissitus ruthenicus* in 1976 and renamed *Meliotoides ruthenica* in 1982 (Soják, 1982). In the *Flora of China* (jointly published by China and the United States in 2010), *M. ruthenica* was reclassified as *Medicago* with the scientific name *Medicago ruthenica* (Wu et al., 2010). According to *Flora of China*, *M. ruthenica* includes one typical species and three varieties: *M. ruthenica* (*M. ruthenica* [L.] Soják), *Yinshan M. ruthenica* (*M. ruthenica* var. inshanicus), *Liaoxi M. ruthenica* (*M. ruthenica* var. liaosiensis), and *fine-leaved M. ruthenica* (*M. ruthenica* var. oblongifolia). Through integration of previous findings, the genetic separation between *M. sativa* and *M. ruthenica* may be more than that between *M. sativa* and *Medicago* edgeworthii (Campbell and Bauchan, 2002). However, precise classification of *M. ruthenica* requires additional molecular evidence.

Genome analysis has significant implications for crop phylogeny, morphology, and breeding. Chloroplasts and mitochondria are semiautonomous eukaryotic organelles that contain DNA. According to endosymbiotic theory, mitochondria and chloroplasts originated from free-living organisms, with an increasing number of genes being transferred to the nuclear genome over evolutionary time (Berry, 2003). These organelles contain essential genes for respiration and photosynthesis. In contrast to animal mitochondrial genomes, plant mitochondrial genomes exhibit remarkable variability and complexity in terms of genome size, gene content, gene arrangement, RNA editing, gene loss, and DNA sequence transfer, and are characterized by complex combinations, diverse structures, variable non-coding regions, and highly recombinogenic repetitive sequences (Kempken, 2011). Mutations in non-coding intergenic regions are repaired via non-homologous end-joining or break-induced replication, resulting in genomic expansion, rearrangements, and chimerism. (Wang et al., 2024a). Repetitive sequences also influence the complexity of plant mitochondrial genomes, such as the presence or absence of inverted repeats were selected by species in changing environments (Christensen, 2013; Wu et al., 2015). *Medicago* species are usually considered as an inverted repeat (IR) lacking clade (IRLC) legume species that have been found newly regrown IR sequence during evolution (Choi et al., 2020). Plant mitochondrial genomes frequently undergo rearrangement and expansion: they can eliminate inaccurately repaired coding regions through natural selection, whereas inaccurately repaired non-coding regions tend to persist (Christensen, 2014). Back-mapping of long reads to these repeats verified the different conformations for each accession and that the varied conformations did indeed exist simultaneously. The existence of these various conformations and structural variations in *Morus* mitogenomes may influence their evolutionary dynamics, and their ability to adapt to environmental changes, and can also offer increased flexibility for interactions with the nuclear genome (Liu et al., 2024).

Besides, even among closely related species, mitochondrial genomes display substantial variation in size, structure, and genetic content (Kubo and Newton, 2008). In terms of synonymous substitution rate, compared to the rapidly evolving nuclear genome and the second most rapidly evolving chloroplast genome, mitogenomes evolve slowly in sequence (Wang et al., 2024b). Consequently, mitochondrial genome data have been widely applied in plant phylogenetic systematics and taxonomic studies (Ruprecht et al., 2017), including those on the gramineous forage *Elymus sibiricus*, legume forage *Medicago sativa* ssp. *sativa*, and medicinal plants such as *Carthamus tinctorius*, *Solanum tuberosum*, *Sorghum bicolor*, and *Glycine max* (Guisinger et al., 2010; Liu et al., 2021; Xiong et al., 2022; He et al., 2023; Wu et al., 2023; Zhang et al., 2023). Distinct types of mutation events in mitochondrial genomes enable species identification and evolutionary branch reconstruction across different timescales (Wang et al., 2021). Therefore, a comprehensive analysis of lentil (*Lens culinaris* Medik) mitochondrial genome can provide critical scientific evidence for refining its taxonomic placement.

The mitochondrial genomes of legume plants exhibit significant structural and functional diversity, providing abundant resources for studying the evolutionary relationships and genetic characteristics among these species. *M. ruthenica* is an important protein-rich forage crop belonging to the legume family. Studies of its nuclear and chloroplast genomes have revealed unique adaptive mechanisms and evolutionary traits under environmental pressure. The nuclear genome of *M. ruthenica* spans 903.56-904.13 Mb and contains approximately 50,162-50,268 protein-coding genes (PCGs). This gene number is larger than that in related species such as *Medicago truncatula* and *M. sativa* ssp. *sativa*, primarily due to repetitive element expansion (Yin et al., 2021). Phylogeographic analyses identified two distinct genetic lineages in *M. ruthenica* consistent with its east-west distribution. These lineages likely diverged during the Last Glacial Maximum and persisted in multiple refugia (Yan et al., 2021). Chloroplast genome phylogenies indicate a close relationship between *M. ruthenica* and *M. truncatula* (Xie et al., 2021). However, cp genome annotations for *M. ruthenica* vary significantly, with reported gene counts of 107, 108, and 116 genes (Melton et al., 2015; Keller et al., 2017; Xu et al., 2021). Such discrepancies may arise from extensive structural rearrangements and gene losses in plant chloroplast genomes, potentially compromising the accuracy of phylogenetic inferences. The current taxonomic evidence for *M. ruthenica* remains inconclusive (Semerikov et al., 2022).

Mitochondrial genomes have been widely used to reconstruct phylogenetic relationships, shedding light on species’ geographical distributions and migration histories (Arseneau et al., 2017). Mining functional sequence information is crucial for exploring plant phylogenies relationships. In the present study, we sequenced and annotated the mitochondrial genome of *M. ruthenica* and conducted comprehensive analyses focusing on genomic characteristics, repetitive sequences, and codon usage bias. Phylogenetic analysis was performed to clarify the genetic divergence in *M. ruthenica* in combination with existing reports on its breeding and plant research. Our findings provide a theoretical foundation for future research.

## 2 Materials and methods

### 2.1 DNA extraction, genome sequencing, and assembly

Fresh leaves of *M. ruthenica* were collected from Hohhot, Inner Mongolia, China (40.57°N, 111.93°E), and stored at the National Perennial Forage Germplasm Resource Nursery in Hohhot, China. Genomic DNA was isolated from fresh leaves using the Plant DNA Isolation Kit (Tiangen, Beijing, China). In this study, we first performed sequencing using the Novaseq6000 (Illumina, San Diego, CA, USA) and PromethION (Oxford Nanopore Technologies, Oxford, UK) platforms. For mitochondrial genome assembly, the Nanopore long-read data were initially aligned against reference gene sequences (https://github.com/xul962464/plant_mt_ref_gene) using minimap2 (v2.1) (Li et al., 2016). Subsequently, Canu (v2.0) was used for error correction and Bowtie2 (v2.3.5.1) was employed to align the second-generation data with the corrected sequence (Koren et al., 2017; Langmead and Salzberg, 2012). Finally, Unicycler (v0.4.8) with default parameters was employed to merge the matched second-generation data with the corrected third-generation data, aiming to obtain a complete and accurate mitochondrial genome sequence (Wick et al., 2015). Using Bandage (v0.8.1), we successfully assembled the mitochondrial genome of *M. ruthenica*. To ensure the reliability of the assembled mitochondrial genome, we conducted a comprehensive validation through coverage depth analysis using raw sequencing data from both NovaSeq 6000 and PromethION platforms (BioProject: PRJNA1291276; BioSample: SAMN49959260; SRA (Illumina): SRR34532195; SRA (ONT): SRR34532194). For the NovaSeq 6000 short-read data, we performed reference sequence alignment using Bowtie2 (v2.3.5.1) (Langmead and Salzberg, 2012). In parallel, we processed Oxford Nanopore long-read data through reference-guided assembly with minimap2 (v2.1) (Li et al., 2010). Both alignment procedures were complemented by SAMtools (v1.9) for in-depth coverage analysis and quality assessment throughout the entire mitochondrial genome sequence (Li et al., 2009). Sequences of the *M. ruthenica* mitochondrial genome were deposited in the GenBank (accession number: PP723040).

### 2.2 Genome annotation

To annotate the PCGs in the *M. ruthenica* mitochondrial genome, we used the whole genomes of six previously published alfalfa species (*Medicago polymorpha* MW971561, *M. sativa* ssp. *sativa* NC_068105, *Medicago sativa* ssp. *falcata* OQ612687, *M. truncatula* NC_029641, *Medicago arabica* OX326972 and *Medicago lupulina* OY283147) as a reference. Genome annotation was performed using Geseq (v2.03) and PMGA (Li et al., 2025; Lowe and Eddy, 1997). tRNA and rRNA genes were verified with tRNAscan-SE (v2.0.12) using default parameters (Greiner et al., 2019), while open reading frames (ORFs) of ≥ 300 base pairs (bp) were identified via ORFfinder (https://www.ncbi.nlm.nih.gov/orffinder/). A circular mitochondrial genome map was generated using Organellar Genome DRAW (v1.3.1) (Beier et al., 2017).

### 2.3 Repeat sequence identification

The *M. ruthenica* mitogenome was analyzed for repeats-simple sequence repeats (SSRs, also called microsatellite) motifs using MISA (v2.1) (Benson, 1999), with detection thresholds set to mono-10, di-5, tri-4, tetra-3, penta-3, and hexa-3 repeats. Tandem Repeats Finder (v4.09) was applied with default parameters to identify tandem repeats (Kurtz, 2001), while dispersed repeats of ≥ 70 bp were characterized as forward, reverse, palindromic, and complementary repeats using REPuter (Iriarte et al., 2021). The analysis parameters included a Hamming distance of 3 and e-value cut-off of 1e-5.

### 2.4 Codon usage bias analysis

Codon (v1.4.4) software was used to identify the codon usage of the mitochondrial genomes of *M. ruthenica*, *M. truncatula*, *M. polymorpha*, *M. sativa* ssp*.falcata*, *M. sativa* ssp*.sativa*, *M. arabica* and *M. lupulina* (Peden, 2000). First, perl scripts were used to extract PCGs with a length greater than 300 bp, starting with ATG and terminating with TAA, TAG or TGA, while removing duplicate genes. Subsequently, the standard protein coding sequence (CDS) was input into Codon (v1.4.4) software in FASTA format, and the relative synonymous codon usage (RSCU) was calculated. The histogram was drawn using the R language ggplot2 package (Wickham, 2009).

### 2.5 Prediction of RNA editing sites

Based on six RNA-seq datasets of *M. ruthenica* deposited in the Sequence Read Archive (SRA, https://www.ncbi.nlm.nih.gov/sra/) under accession numbers SRR4140266, SRR4140268, SRR4140269, SRR4140270, SRR4140271, and SRR4140272, we established putative RNA editing sites in the mitochondrial PCGs. The alignment of RNA sequences for mitochondrial PCGs was performed using BWA (v0.7.15) (Li and Durbin, 2010). SNP identification was subsequently carried out with SAMtools (v1.17) and BCFtools (v1.17) (Li et al., 2009; Danecek et al., 2021). RNA editing sites were detected and annotated using REDO (v1.0) with its default parameters (Wu et al., 2018). To eliminate false editing sites, DNA-seq data were aligned to the *M. ruthenica* mitochondrial genome using BWA, BCF tools were used to identify genomic SNPs and exclude overlapping sites from RNA editing candidates.

### 2.6 Chloroplast-derived mitochondrial sequence identification

The chloroplast genome of *M. ruthenica* was *de novo* assembled using GetOrganelle (v1.7.5.3) software and performed annotation with PGA (Jin et al., 2020; Qu et al., 2019). Homologous fragments in the mitochondrial and chloroplast genomes were identified using BLASTn (v2.14.1+) (Chen et al., 2015), and gene transfer from the chloroplasts to mitochondria was visualized using TBtools-II (v2.136) (Chen et al., 2020).

### 2.7 Selective pressure calculation (Ka/Ks)

MAFFT (v7.526) was used for multiple sequence alignment of 26 common genes (*atp*1, *atp*4, *atp*6, *atp*8, *atp*9, *ccmB*, *ccmC*, *ccmFc*, *cob*, *cox*1, *cox*3, *matR*, *matB*, *nad*1, *nad*2, *nad*3, *nad*4, *nad*4L, *nad*5, *nad*6, *nad*7, *nad*9, *rpl*16, *rp*l5, *rps*12, *rps*4) between the mitogenomes of *M. ruthenica* and 33 other legume species (Katoh et al., 2019). Then import Ka/Ks Calculator (2.0) software (Wang et al., 2010), select the Yang-Nielsen (YN) model to calculate the non-synonymous substitution rate (Ka) and the synonymous substitution rate (Ks), and further calculate the Ka/Ks ratio. Finally, the Ka/Ks value box plot is drawn through the R language ggplot2 package.

### 2.8 Nucleotide diversity (Pi) analysis

Based on 30 common genes (*atp1*, *atp4*, *atp6*, *atp8*, *atp9*, *ccmB*, *ccmC*, *ccmFc*, *ccmFn*, *cob*, *cox1*, *cox2*, *cox3*, *matR*, *matB*, *nad1*, *nad2*, *nad3*, *nad4*, *nad4L*, *nad5*, *nad6*, *nad7*, *nad9*, *rpl16*, *rpl5*, *rps12*, *rps14*, *rps4* and *rps7*) in the mitochondrial genomes of *M. ruthenica*, *M. truncatula*, *M. polymorpha*, *M. sativa* ssp*.falcata*, *M. sativa* ssp*.sativa*, *M. arabica* and *M. lupulina*. MAFFT (v7.526) was used for alignment, and then the alignment sequence was input into DnaSP (v6.12.03) for Pi calculation (Rozas et al., 2017).

### 2.9 Phylogenetic analysis

Phylogenetic analysis was performed using 18 shared PCGs (*atp4*, *atp8*, *ccmC*, *cob*, *cox1*, *cox3*, *matR*, *nad2*, *nad3*, *nad4*, *nad5*, *nad6*, *nad7*, *nad9*, *rpl16*, *rps12*, *rps3* and *rps4*) from 34 legume species mitogenomes. *A.* sp*eltoides* and *A. thaliana* were designated as the outgroup taxa. The common genes were extracted using the PhyloSuite (v1.2.3) software, and aligned with MAFFT (v7.526) (Zhang et al., 2020). Disordered positions and divergent regions were removed using Gblocks 0.91b (Talavera and Castresana, 2007). The Model Finder tool in PhyloSuite v1.2.3 (Kalyaanamoorthy et al., 2017; Zhang et al., 2020) was used to select optimal partitions for Bayesian and ML constructions. Subsequently, the MrBayes (v3.2.7) software was used to construct a Bayesian Inference (BI) tree (Ronquist et al., 2012), with the optimal model being GTR+F+I+G4. RAxML (v8.2.12) was employed to construct the maximum likelihood (ML) tree with 1000 bootstrap replicates (Stamatakis, 2006), and the optimal partition model was set as GTRGAMMAI.

### 2.10 Collinearity analysis

The mitochondrial genome sequences of seven species of *M. ruthenica* and *M. truncatula*, *M. polymorpha*, *M. sativa* ssp*. falcata*, *M. sativa* ssp*. sativa*, *M. arabica* and *M. lupulina* were analyzed for covariance. The homologous sequences of these seven species were first aligned two-by-two based on BlastN (v2.13.0) to filter out the high-confidence homologous regions with a length of more than 300 bp (Chen et al., 2015). These segments were then analyzed for multiple covariance using MCScanX and plotted for multiple covariance mapping (Wang et al., 2012).

## 3 Results

### 3.1 Genomic characteristics of the mitochondrial genome in *M. ruthenica*

**Figure 1:**
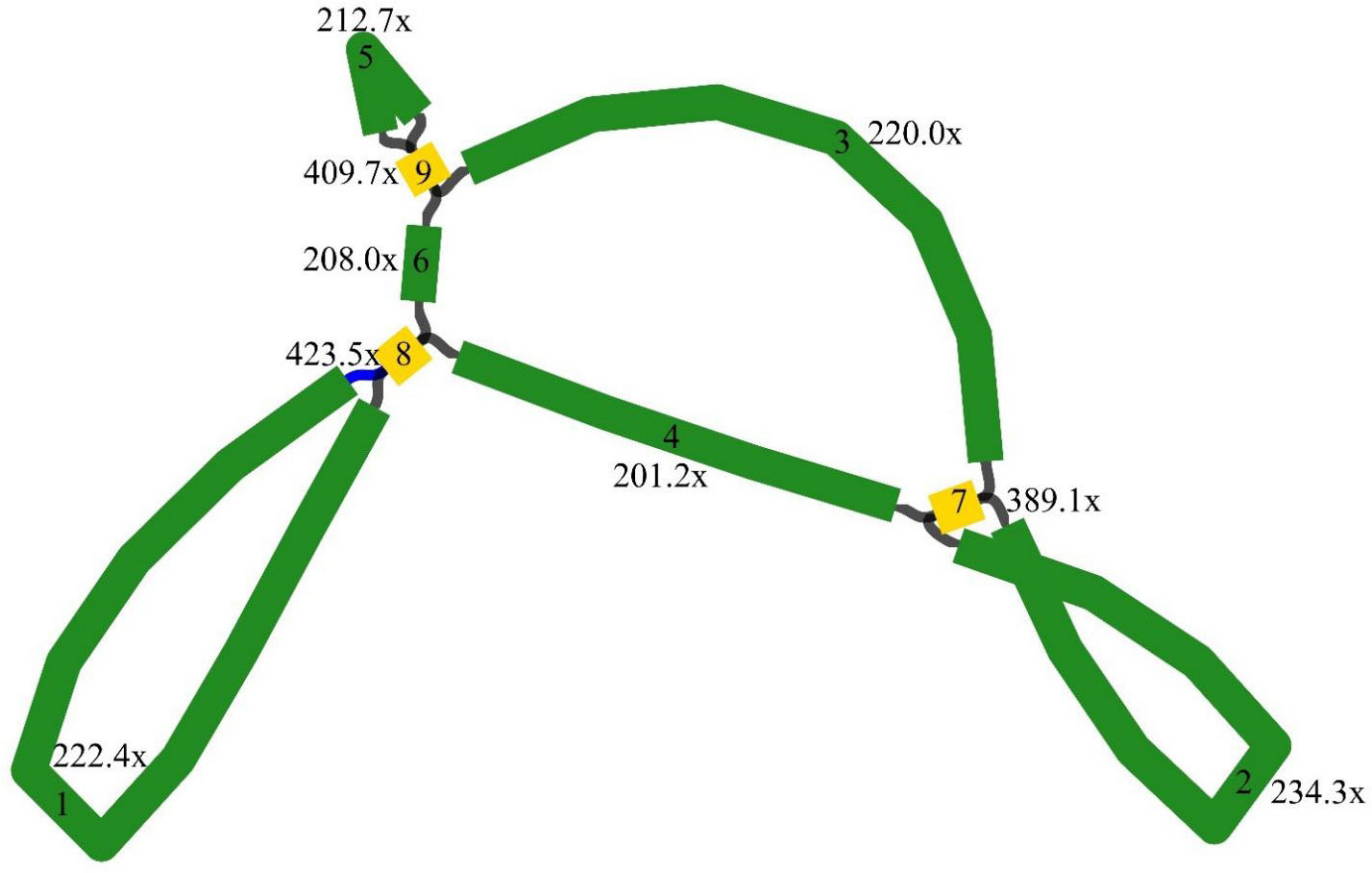
Genome assembly graph of the *Medicago* mitogenome. Nodes (yellow squares) represent genomic segments flanking repeats, edges (green segments) denote potential connections between these segments, the distinct coverage values of edges indicate differential connectivity patterns within repetitive regions.

**Figure 2:**
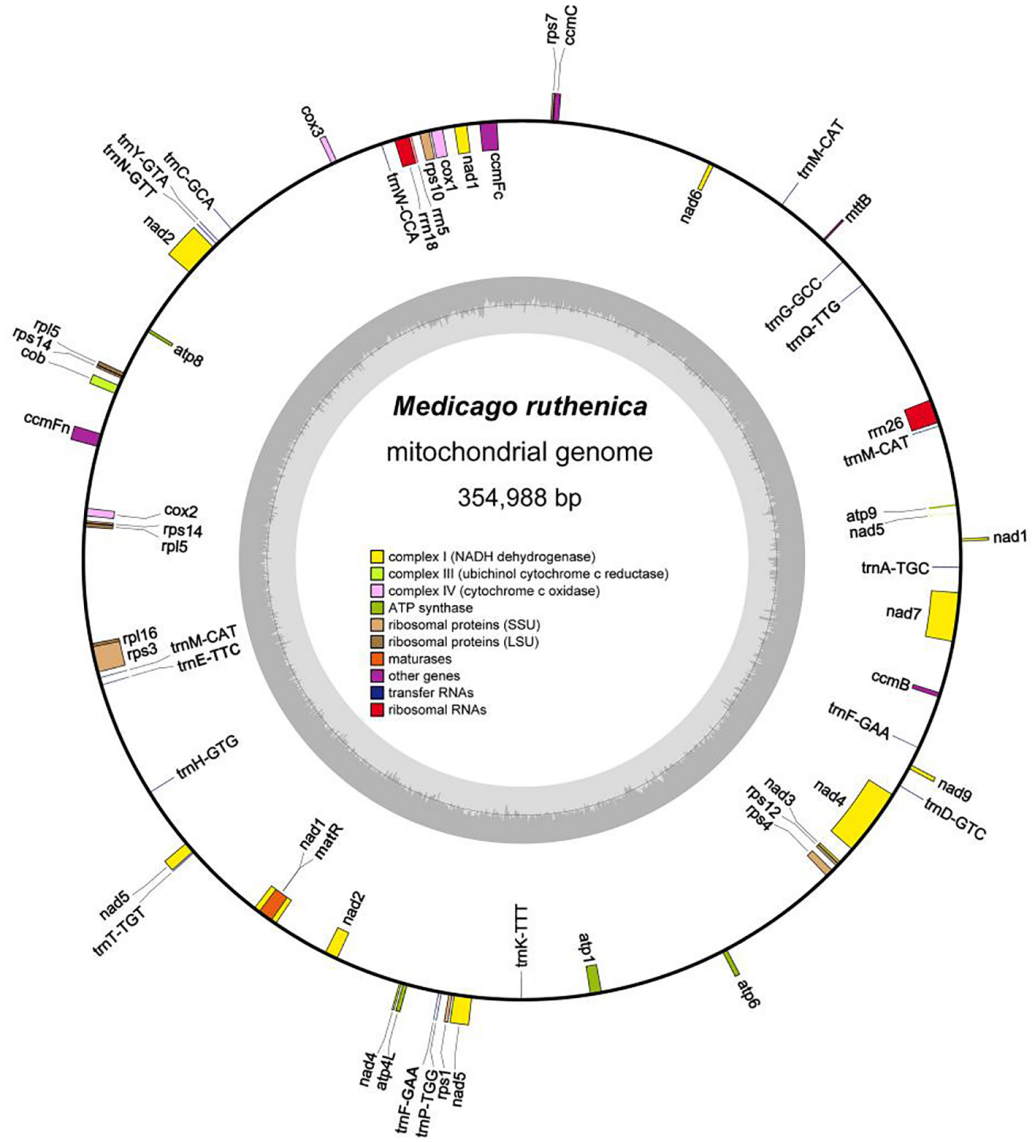
Circular diagram representing the complete mitochondrial genome of *M. ruthenica* and illustrating the gene organization. Genes positioned on the outer side of the circle are transcribed in the forward direction, while those on the inner side are transcribed in the reverse direction. The inner charcoal grey region indicates the GC content. Genes belonging to different functional categories are distinguished using various colors.

The *M. ruthenica* mitogenome assembly graph revealed differences in the distribution, connection, and coverage of repetitive sequences. Sites eight and nine harbored inversions, while site seven formed a macrocycle or two small subcycles (**Figure 1**). It was assembled into a single circular-mapping molecule of 354,988 bp (**Figure 2**). With a GC content of 45.13%, the mitogenome had a higher percentage than its AT content of 54.87% (**Supplementary Table S1**). Within the genome, the CDS spanned 29,844 bp, with 42.76% GC and 57.24% AT content (**Supplementary Table S2**). Transfer RNA (tRNA) genes constituted 1,309 bp with 51.57% GC and 48.43% AT contents, whereas ribosomal RNA (rRNA) genes spanned 5,239 bp with 51.67% GC and 48.33% AT contents. These results indicate that the GC content of protein-coding sequences are lower than that of tRNA genes.

*Nad1*, *nad2*, *nad5*, and *nad9* each contained four introns (**Supplementary Table S3**); *nad4* contained three; and the remaining genes lacked introns. Ribosomal protein genes spanned a total length of 5,994 bp; *rps1* was designated as a pseudogene, *rpl5* and *rps14* each had two copies, and only *rps10* and *rps3* contained one intron (**Table 1**). The three longest genes were *nad5* (2,010 bp), *matR* (1,986 bp), and *ccmFn* (1,737 bp), and the shortest was *atp9* (225 bp). A total of 18 tRNA and three rRNA genes were randomly interspersed among the PCGs. Not all genes were located on the positive strand; 12 tRNA genes and all rRNA genes were located on the negative strand. Only *trnA-TGC* and *trnT-TGT* contained one intron each, whereas *trnF-GAA* and *trnM-CAT* contained two or three copies.

**Table 1:** Start and stop codon information of the *M. ruthenica* mitochondrial gene.

Gene Category	Group of genes	Gene name
Core genes	ATP synthase	*atp1*, *atp4*, *atp6*, *atp8*, *atp9*
	Cytochrome c biogenesis	*ccmB*, *ccmC*, ccmFc*, *ccmFn*
	Ubiquinol cytochrome c reductase	*cob*
	Cytochrome c oxidase	*cox1*, *cox2*, *cox3*
	Maturases	*matR*
Variable genes	Transport membrane protein	*mttB*
	NADH dehydrogenase	nad1****, nad2****, *nad3*, nad4***, *nad4L*, nad5****, *nad6*, nad7****, *nad9*
	Ribosomal proteins (LSU)	*rpl16*, *rpl*5(2)
	Ribosomal proteins (SSU)	*#rps1*, rps10*, *rps12*, *rps14*(2), rps3*, *rps4*, *rps7*
rRNA genes	Ribosomal RNAs	*rrn18*, *rrn26*, *rrn5*
tRNA genes	Transfer RNAs	trnA-TGC*, *trnC-GCA*, *trnD-GTC*, *trnE-TTC*, *trnF-GAA*(2), *trnG-GCC*, *trnH-GTG*, *trnK-TTT*, *trnM-CAT*(3), *trnN-GTT*, *trnP-TGG*, *trnQ-TTG*, trnT-TGT*, *trnW-CCA*, *trnY-GTA*

*: ntron number; #Gene: pseudo gene; Gene (2): number of copies of multi-copy genes.

### 3.2 Repetitive sequences in the *M. ruthenica* mitochondrial genome

**Figure 3:**
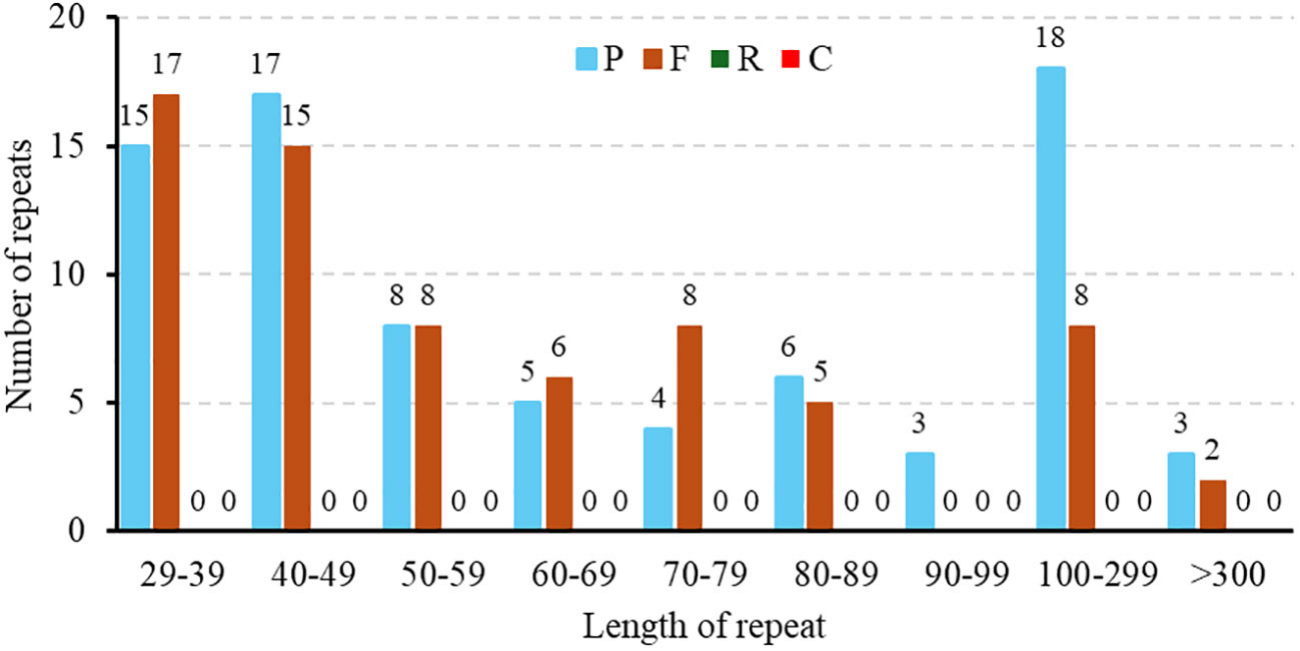
Distribution of dispersed repeat lengths in the *M. ruthenica* mitochondrial genome. The X-axis represents the categories of dispersed repeats, while the Y-axis shows the quantity of scattered repeats.

**Figure 4:**
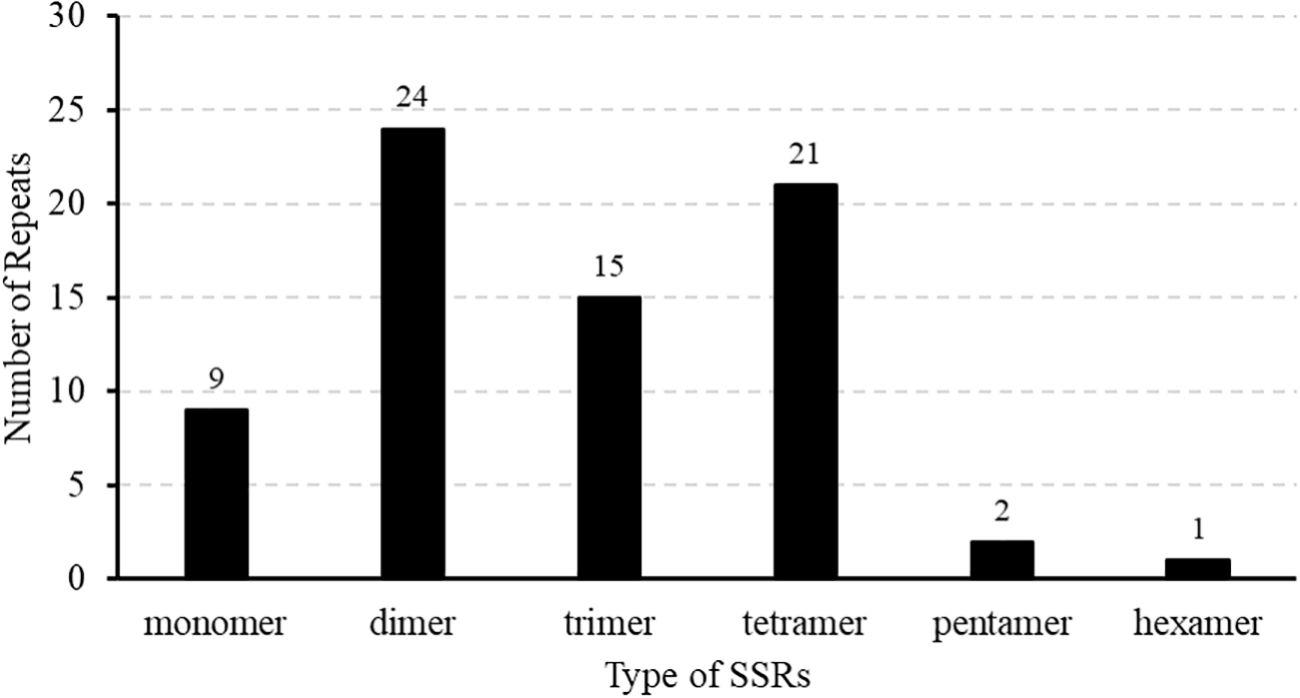
Patterns of SSR distribution in the *M. ruthenica* mitochondrial genome. The X-axis represents the different types of SSRs, while the Y-axis denotes the frequency of SSR repeats.

Repeat elements such as SSRs often play crucial roles in genome variation, evolution, stability, and organization. In this study, repetitive sequence analysis of the *M. ruthenica* mitogenome was performed to explore its structural complexity. In total, 148 repeat sequences with lengths ranging from 29 to over 300 bp were identified in the mitochondrial genome, including 69 forward repeats (F) and 79 palindromic repeats (P) (**Figure 3**). Palindromic and forward repeats accounted for 53% and 47%, respectively. Specifically, 40 forward and 40 palindromic repeats were < 60 bp, 8 forward and 18 palindromic repeats measured 100-300 bp, and two forward and three palindromic repeats exceeded 300 bp. In contrast, the 90-99 bp range was the least frequent, containing only three palindromic repeats. This distribution highlights preferential accumulation of small repeat elements in the *M. ruthenica* mitochondrial genome. In this study, 72 SSRs were identified in the mitochondrial genome of *M. ruthenica* through our analysis (**Figure 4**), revealing diverse compositions. These SSRs comprised nine mononucleotide repeats (12.50%), 24 dinucleotide repeats (33.33%), 15 trinucleotide repeats (20.83%), 21 tetranucleotide repeats (29.17%), two pentanucleotide repeats (2.78%), and one hexanucleotide repeat (1.39%).

Most small repeats were derived from nonfunctional regions of the *M. ruthenica* mitochondrial genome, such as the intergenic spacers (IGSs), totaling 742 bp in repeat length (**Supplementary Table S4**). Specifically, six SSRs were identified in the IGS between *atp1* and *atp6*, including dinucleotide (AG, GT, GA), trinucleotide (GGT), and tetranucleotide (TAAA, ATTA) repeats spanning 72 bp. Six SSRs were detected in the IGS between *trnE-UUC* and *trnH-GUG*, consisting of mononucleotide (T), dinucleotide (AG, TA), trinucleotide (AGT, CTT), and tetranucleotide (TAGA) repeats covering 72 bp. Two SSRs were found in the IGS between *trnT-UGU* and *nad1*, including mononucleotide (A), dinucleotide (TA, CT), and tetranucleotide (AAAT) repeats, totaling 42 bp. These three regions exhibited the highest densities of repeat types. In the intronic regions, five dinucleotide (TC) repeats were identified in *nad2* intron; *nad4* intron contained ten mononucleotide (C), five dinucleotide (AG), and four trinucleotide (AAG) repeats; and *nad5* intron harbored dinucleotide (AG) and trinucleotide (ATA) repeats spanning 64 bp. Only 2 SSRs were detected in the coding regions: three tetranucleotide (AATG) repeats in *rps3* and ten mononucleotide (A) repeats in *nad1*, totaling 22 bp.

A striking feature was the prevalence of A/T-rich SSRs (**Supplementary Table S5**), with 71 (98.61%) SSRs exhibiting A/T richness, including 24 SSRs composed exclusively of A/T bases. These included eight mononucleotide repeats (A/T), nine dinucleotide repeats (AT/TA), three trinucleotide repeats (AAT/TTA, TAT/ATA), and four tetranucleotide repeats (AAAT/ATTT, AATT/TTAA). The abundance of SSRs offers a rich resource for the development of molecular markers that are valuable for identifying and genetically characterizing *M. ruthenica.*

**Figure 5:**
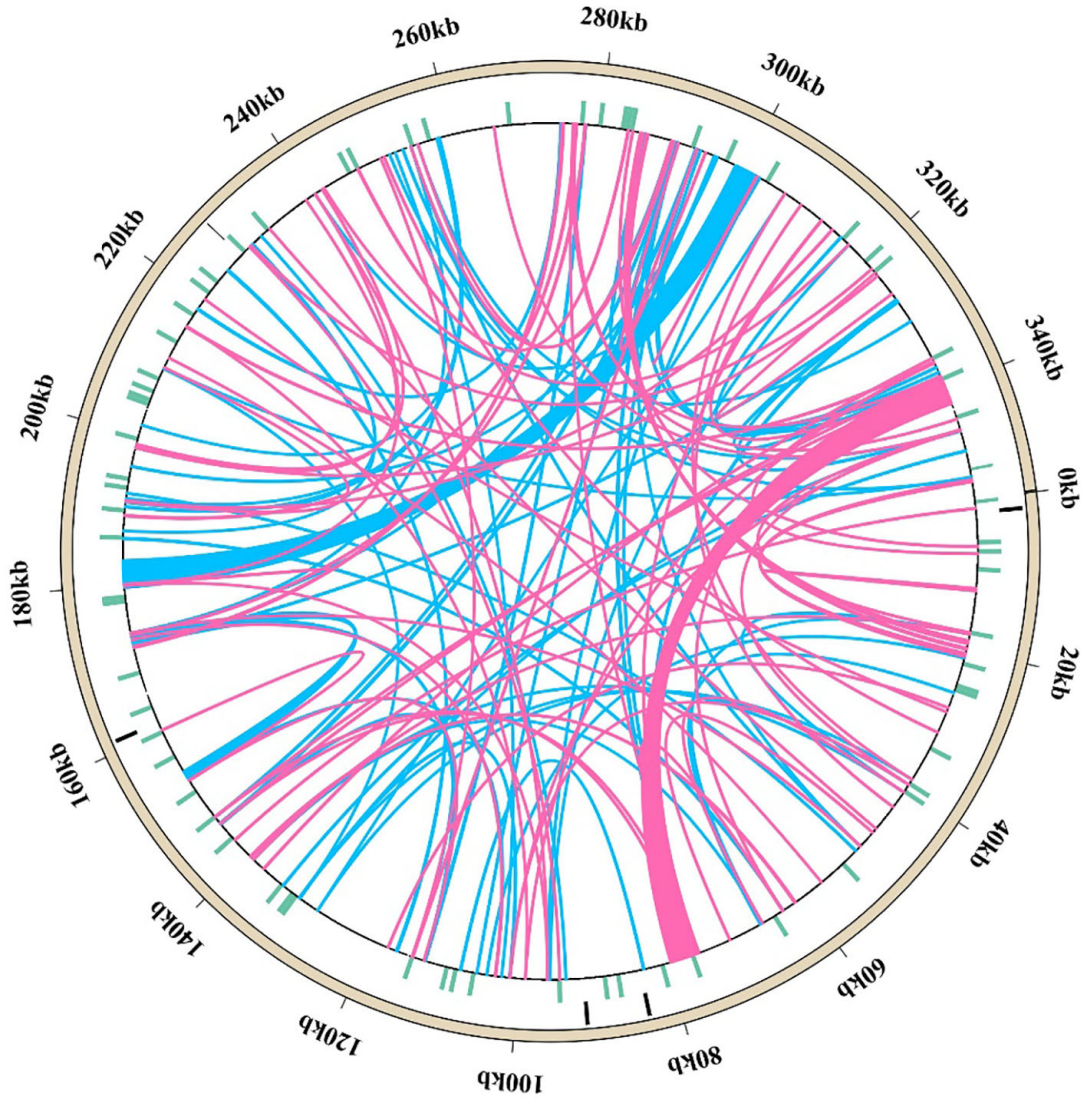
Arrangement of dispersed repeats within the mitochondrial genome of *M. ruthenica*. The outermost ring depicts the mitochondrial genome sequence, followed by layers representing SSR repeats, tandem repeats, and dispersed repeats (pink arcs indicate 69 forward repeats, while blue arcs represent 79 palindromic repeats).

The *M. ruthenica* mitogenome contains 148 dispersed long repeat pairs (≥ 30 bp), including 69 forward and 79 palindromic repeats (**Figure 5**, **Supplementary Table S6**). Among these, 8 forward and 18 palindromic repeats measured 100-300 bp, one forward repeat spanned 400-500 bp, and three palindromic repeats and one forward repeat exceeded 3,000 bp. One pair of forward repeats and one pair of palindromic repeats are localized in the coding region (*atp1*, *cox2*; *matR*, *ccmFc*). One pair of palindromic repeats were localized to the tRNA genes, *trnF-GAA* and *trnF-GAA*. Three pairs of forward repeats and five pairs of palindromic repeats were localized to the introns. Four pairs of forward repeats and five pairs of palindromic repeats were localized in both the intron and the IGS regions. We identified 18 pairs of forward repeats and 12 pairs of palindromic repeats localized in both the coding and the IGS regions, while the others were all found in the intergenic spacer regions. In addition, 11 pairs of forward repeats and 14 pairs of palindromic repeats were localized in the IGS regions between *atp1* and *atp6*. Moreover, 4 pairs of forward repeats and 12 pairs of palindromic repeats were localized in the IGS regions between *atp6* and *rps4*. Finally, three pairs of forward repeats and two pairs of palindromic repeats were localized in the IGS region between *nad2* and *atp8*.

Five tandem repeats (24-41 bp) were detected in the *M. ruthenica* mitogenome (**Supplementary Table S7**). One tandem repeat (CACCTGCAGTTTGGTGCAG) was located in the coding region of *ccmFn*. The remaining repeats resided in the IGS regions, including an 11 bp repeat (TTTCTTTTTCT) between *nad6* and *ccmC*, 12 bp repeat (TACGTATATAAA) between *trnA-TGC* and *nad1*, 19 bp repeat (TTCCAGTTCTGTTATAATA) between *trnT-UGU* and *nad1*, and 21 bp repeat (ATAATTAGTGAACTTATGAGA) between *rps7* and *ccmFc*. These insights into the mitogenome structural dynamics and repeats with potential functional implications enhance our understanding of *M. ruthenica* genome regulation and evolution.

### 3.3 Codon usage bias in PCGs

**Figure 6:**
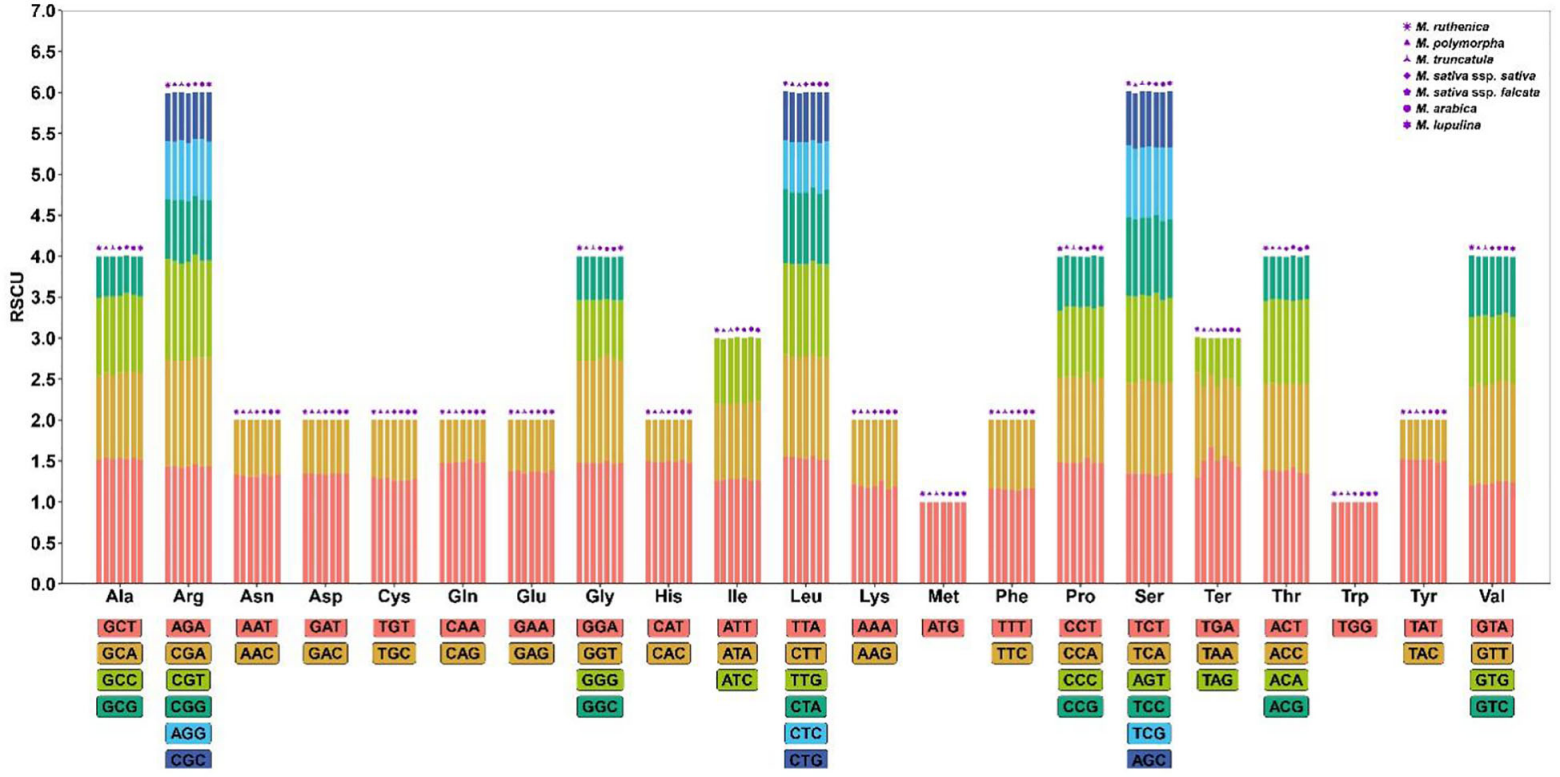
Relative synonymous codon usage (RSCU) of seven *Medicago* mitogenome. Codon families are on the X-axis. RSCU values are the number of times of a particular codon, relative to the number of times that the codon would be observed for a uniform synonymous codon usage.

In this study, the codon usage characteristics of mitochondrial genomes of *M. ruthenica*, *M. truncatula*, *M. polymorpha*, *M. sativa* ssp*. falcata*, *M. sativa* ssp*. sativa, M. arabica* and *M. lupulina* were analyzed (**Figure 6**, **Supplementary Table S8**). The results showed that all species followed the standard genetic framework of 21 amino acids corresponding to 64 codons. Methionine (Met) and Tryptophan (Trp were only encoded by a single codon, while Arginine (Arg), Leucine (Leu) and Serine (Ser) had the most synonymous codons (six each). The RSCU analysis showed that 31 codons in *M. truncatula*, *M. polymorpha*, *M. sativa* ssp*. falcata*, *M. lupulina* and *M. sativa* ssp*. sativa* showed preference (RSCU > 1), 31 codons were non-preference (RSCU < 1), and codons AUG and UGG showed neutral selection characteristics (RSCU = 1). In *M. arabica*, 30 codons exhibited preference (RSCU > 1), 31 codons showed non-preference (RSCU < 1), while the codons UGG, UGA, and AUG displayed characteristics of neutral selection (RSCU = 1). It is worth noting that 30 codons of *M. ruthenica* showed preference (RSCU > 1), 32 codons were non-preference (RSCU < 1), and codon UGG and codon AUG showed neutral selection characteristics (RSCU = 1). The UGA terminator (Ter) of *M. ruthenica* showed preference selection characteristics (RSCU = 1.29), while the RSCU of UGA terminator (Ter) of other species showed non-preference (RSCU < 1), which was different from other species. In addition, both *M. ruthenica* and *M. polymorpha* have the largest RSCU value of 1.55, which is the codon UUA encoding Leu. The RSCU values of the codon UAA encoding Ter in *M. truncatula* and *M. sativa* ssp*. falcata* were the largest, which were 1.67 and 1.56, respectively. The codon GCU encoding Ala in *M. sativa* ssp*. sativa* and *M. arabica* had the largest RSCU value of 1.54. The codon GCU encoding Ala in *M. lupulina* had the largest RSCU value of 1.51. The number of codons in the genomes of *M. ruthenica*, *M. truncatula*, *M. polymorpha*, *M. sativa* ssp*. falcata*, *M. sativa* ssp*. sativa*, *M. arabica* and *M. lupulina* were 9159, 9197, 9033, 8226, 9012, 8231 and 8732 respectively. Among the seven species of *Medicago*, the number of codon UUU encoding Phe was the highest (257-341), and the number of codon UAG encoding Ter was the lowest (4-5).

### 3.4 Prediction of RNA editing sites

**Figure 7:**
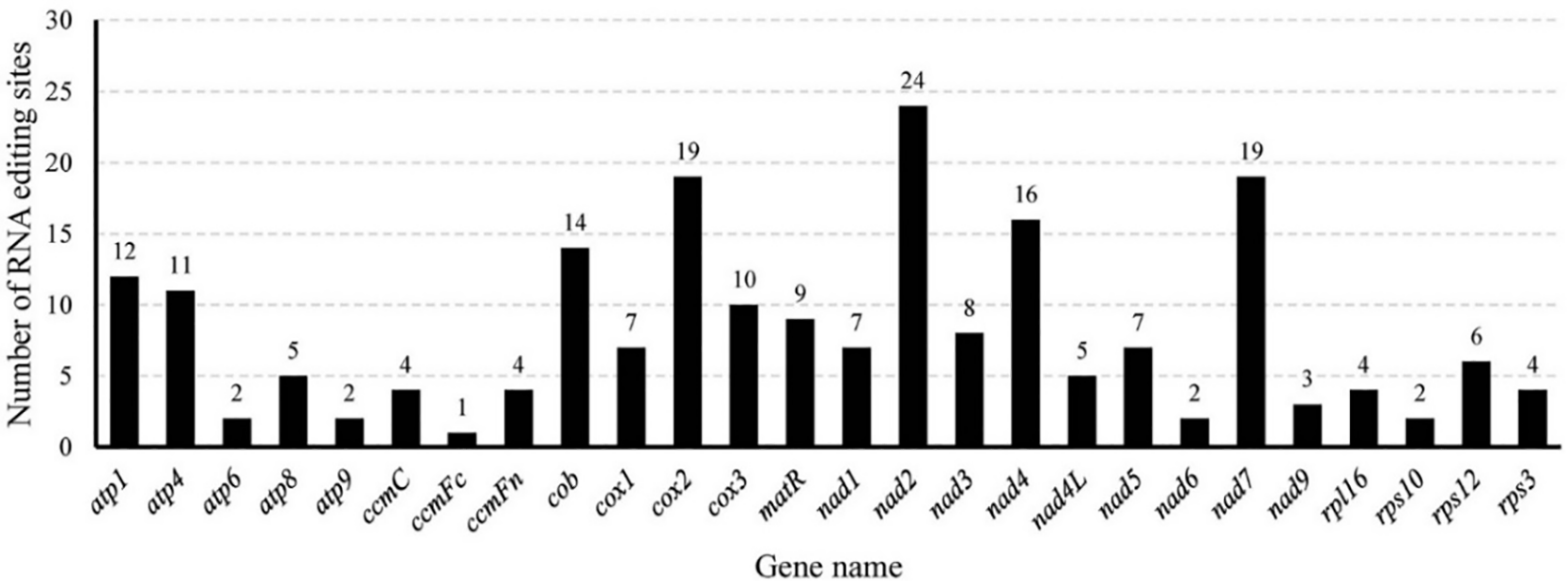
Localization of RNA editing sites in the mitochondrial PCGs of *M. ruthenica*.

Mitochondrial RNA editing is an important post-transcriptional process that affects protein function and stability. A total of 207 RNA editing sites were identified in the 26 PCGs of the *M. ruthenica* mitochondrial genome (**Figure 7**). Among these PCGs, *ccmFc* exhibited the lowest editing frequency with only one editing site, whereas *nad2* was the most frequently edited gene with 24 sites. Both *nad7* and *cox2* displayed intermediate editing levels, with 19 sites each. Almost all RNA editing sites in the *M. ruthenica* mitogenome involved C to C-to-U conversion (**Supplementary Table S9**). Specifically, four C-to-A edits were detected in *cox2* and *nad6*, three G-to-U edits in *atp1* and *nad4L*, four A-to-G or G-to-A edits in *cox2* and *atp1*, one U-to-A edit in *atp1*, one C-to-G edit in *atp1*, and one G-to-C edit in *cox2*. Most RNA editing events occurred at the second nucleotide position (122 events), followed by the first position (71 events), with minimal editing at the third position (14 events). Among these, 100 converted hydrophilic amino acids to hydrophobic residues, 17 converted hydrophobic residues to hydrophilic residues, 18 maintained hydrophilicity, and 71 preserved hydrophobicity.

**Figure 8:**
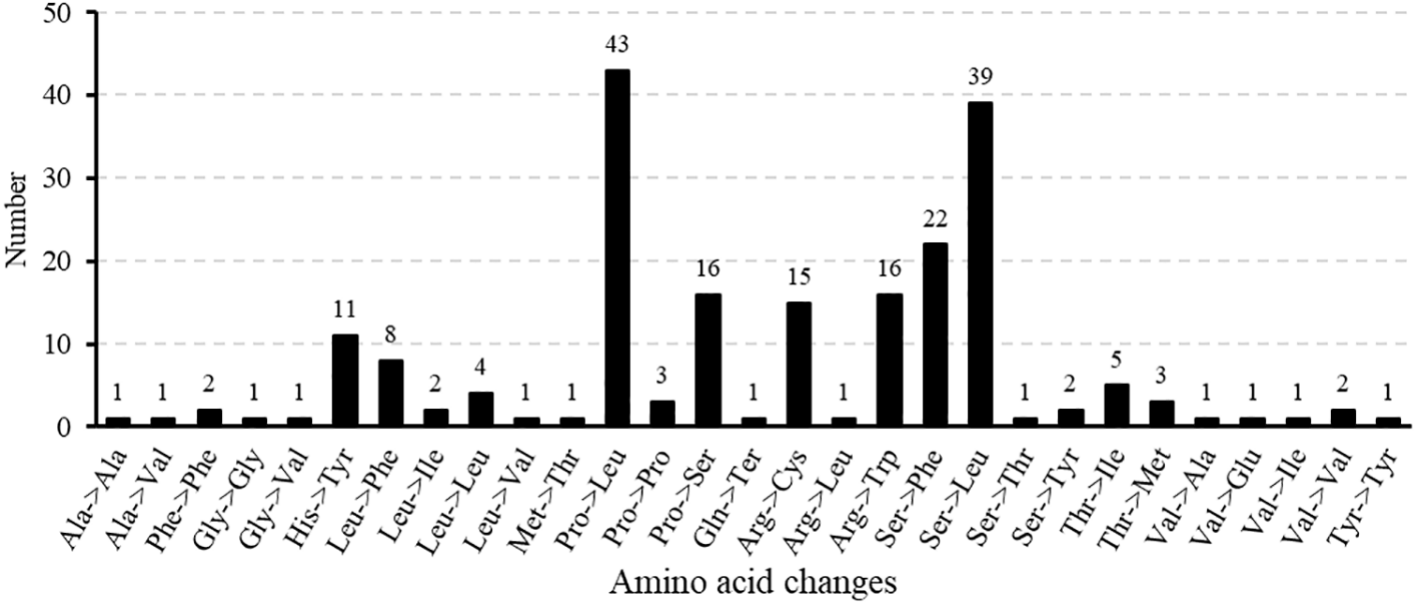
Occurrence of amino acid alterations induced by RNA editing in *M. ruthenica*.

In the *M. ruthenica* mitogenome, an editing event that converts CGA (Gln) to TAG (stop codon) was identified in *rpl16*. Three editing events converting ACG (Thr) to ATG (start codon) were detected in *cox2* and *nad7*, whereas only one event converting ATG (start codon) to ACG (Thr) was detected in *nad4L*. Among the amino acid substitutions, Pro to Leu was the most frequent (43 events), followed by Ser to Leu (39 events), and Ser to Phe (18 events), with other conversions being less common (**Figure 8**).

### 3.5 Chloroplast-originated sequences in the mitochondrial genome

**Figure 9:**
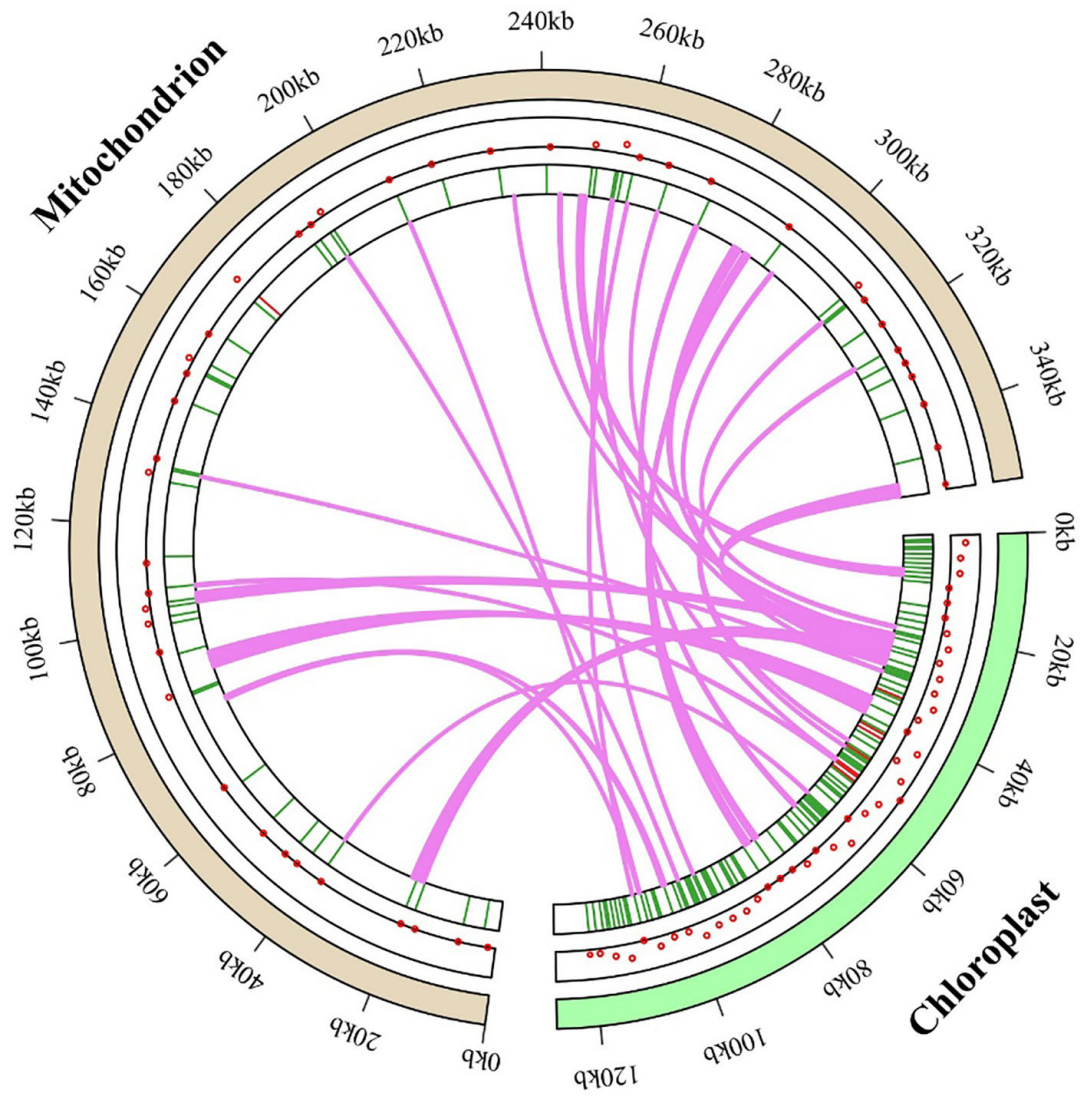
*M. ruthenica* genomic evolution, with gene transfer between chloroplast and mitochondrial genomes depicted using dots and heat maps within circular genome diagrams. Green segments represent the chloroplast genome, purple segments signify the mitochondrial genome, and lawn-green lines trace the pathways and integration sites of chloroplast-derived sequences into the mitochondrial genome. This visualization offers a comprehensive perspective on the dynamics of gene transfer between these organelles.

The *M. ruthenica* mitochondrial genome spans 354,988 bp, approximately 2.80-fold larger than its chloroplast counterpart (126,939 bp). This size difference resulted in a more dispersed mitochondrial gene arrangement compared with that of the compact chloroplast genome (**Figure 9**). Analysis of chloroplast-specific (cp) sequences identified 42 fragments (46-3,546 bp) in the mitogenome, totaling 16,654 bp (4.69% of the mitochondrial genome) (**Supplementary Table S10**). These sequences migrate via diverse pathways: gene-to-gene, gene-to-IGS, IGS-to-IGS, and gene-to-intron/exon pathways. Among them, eight fragments derived from the chloroplast IGS regions were translocated to the mitochondrial IGS regions. Five sequences from chloroplast-encoded genes (*ndhF*, *petG*, *psaA*, *psaB*, *rbcL*) were transferred to the mitochondrial IGS regions, suggesting functional loss and integration into mitochondrial non-coding regions. Additionally, chloroplast *atpA* was converted to mitochondrial atp*1*. Nine migratory sequences from chloroplast tRNA genes (*trnA-UGC*, *trnD-GUC*, *trnE-UUC*, *trnF-GAA*, t*rnH-GUG*, *trnN-GUU*, *trnP-UGG*, *trnM-CAU*, *trnQ-UUG*) were translocated to four mitochondrial IGS regions or five mitochondrial tRNA genes, accounting for 21.43% of all transferred sequences. We identified 12 migratory sequences from chloroplast ribosomal RNA genes (*rrn16* and *rrn23*) that were integrated into mitochondrial IGS regions.

### 3.6 Selective pressure analysis (Ka/Ks) in the mitochondrial genome

**Figure 10:**
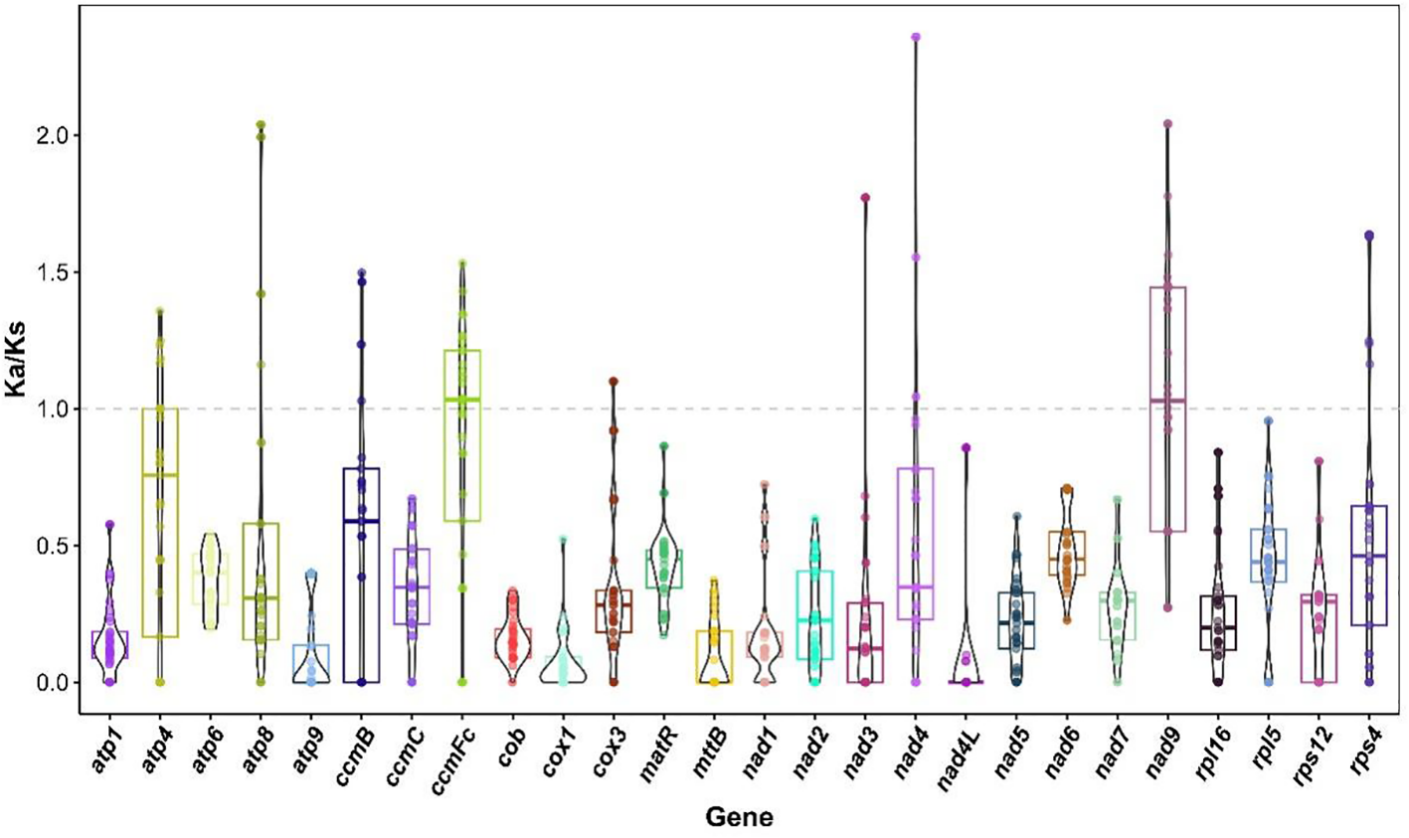
Ka/Ks values of 26 shared genes in mitogenomes of *M. ruthenica* versus 31 legumes species.

In this study, the Ka/Ks values of 26 common genes in the mitochondrial genomes of 34 legume species were analyzed to reveal the selection patterns of these genes during evolution (**Figure 10**, **Supplementary Table S11**). In general, the Ka/Ks values of *cox1*, *cob*, *nad7* and *nad4L* were lower than 0.3 in many species, showing a strong purifying selection signal, indicating that these genes were highly conserved in function. The Ka/Ks values of *atp8*, *atp4*, *rps4*, *nad4*, *ccmB*, c*cmFc* and *nad9* in multiple species were above one, showing obvious positive selection signals. The *ccmFc* showed significant positive selection pressure (Ka/Ks > 1) in *Arachis hypogaea*, *Lupinus albus*, *Apios americana*, *Glycyrrhiza glabra*, *Glycyrrhiza uralensis*, *Ghcine max*, *Gheine soja*, *Astragalus complanatus*, *Gleditsia sinensis*, *Oxytropis* sp*lendens*, *Oxytropis arctobia*, *Lotus japonicus* and *Lotus japonicus*. Among them, the Ka/Ks is the largest in *L. albus*, which is 1.53154. A*. hypogaea*, *L. albus*, *Leucaena trichanda*, *G. sinensis* and *Leucaena leucocephala* showed significant positive selection pressure on *atp8* (Ka/Ks > 1), and the Ka/Ks was the largest in *L. trichanda* and *L. leucocephala*, both 2.03829. In *L. japonicus*, *Senna tora*, *Senna occidentalis*, *L. trichanda* and *M. ruthenica*, *atp4* showed significant positive selection pressure (Ka/Ks > 1), and the Ka/Ks was the largest in *Senna occidentalis*, which was 1.35608. *Rps4* showed significant positive selection pressure (Ka/Ks > 1) in *L. albus*, *Vigna unguiculata*, *Vigna radiata*, *Millettia pinnata*, *G. max* and *G. soja*, and the Ka/Ks was the largest in *G. max* and *G. soja*, both of which were 1.63573. *Nad4* showed significant positive selection pressure (Ka/Ks > 1) in *G. glabra*, *G. uralensis* and *M. ruthenica*, and the Ka/Ks was the highest in *M. ruthenica*, which was 2.35843. *Nad9* showed significant positive selection pressure in *Ammopiptanthus mongolicus*, *Ammopiptanthus nanus*, *L. albus*, *A. americana*, *V. unguiculata*, *G. glabra*, *G. uralensis*, *V. radiata*, *G. max*, *G. soja*, *G. sinensis*, *A. complanatus*, *O.* sp*lendens*, *O. arctobia* (Ka/Ks > 1). Among them, the Ka/Ks was the highest in *O.* sp*lendens*, *A. complanatus* and *O. arctobia*, all of which were 2.04088. The *ccmB* showed significant positive selection pressure (Ka/Ks > 1) in *A. mongolicus*, *A. nanus*, *L. albus*, *A. complanatus*, *O.* sp*lendens* and *O. arctobia*, and the Ka/Ks was the largest in *L. albus*, both of which were 1.49698.

In addition, the Ka/Ks values of *ccmFc*, *cox3*, *atp4*, *nad4*, *nad9* and *ccmB* in some species are close to the neutral evolution threshold (Ka/Ks ≈ 1), suggesting that these genes may be in a state of transition from purification selection to positive selection. The Ka/Ks values of *ccmFc* in *V. unguiculata*, *G. uralensis*, *V. radiata*, *S. occidentalis*, *L. trichanda*, *Trifolium pratense*, *L. leucocephala* and *Pisum fulvum* ranged from 0.976155 to 1.09295. The Ka/Ks value of *cox3* in *P. fulvum* is 1.09941. The Ka/Ks values of *atp4* in *A. hypogaea*, *G. glabra*, *G. uralensis*, *A. complanatus*, *L. leucocephala*, *O.* sp*lendens*, *O. arctobia* ranged from 0.967073 to 1.00096. The Ka/Ks values of *nad4* in *G. max*, *L. trichanda* and *G. soja* ranged from 0.96150 to 1.04318. The Ka/Ks values of *nad9* in *A. hypogaea*, *L. japonicus*, *S. tora* and *S. occidentalis* ranged from 0.968876 to 1.08186. The Ka/Ks value of *ccmB* in *G. sinensis* was 1.02828. It is worth noting that the Ka/Ks values of multiple genes in s*even Medicago* specie*s* were zero or not applicable. Through the above analysis, this study not only comprehensively covers the changes of Ka/Ks different genes in various species, but also highlights the importance of key genes in evolution, which brings new clues to explore the evolutionary mechanism of the mitochondrial genome of legume plants.

### 3.7 Nucleotide diversity analysis (Pi) in the mitochondrial genome

**Figure 11:**
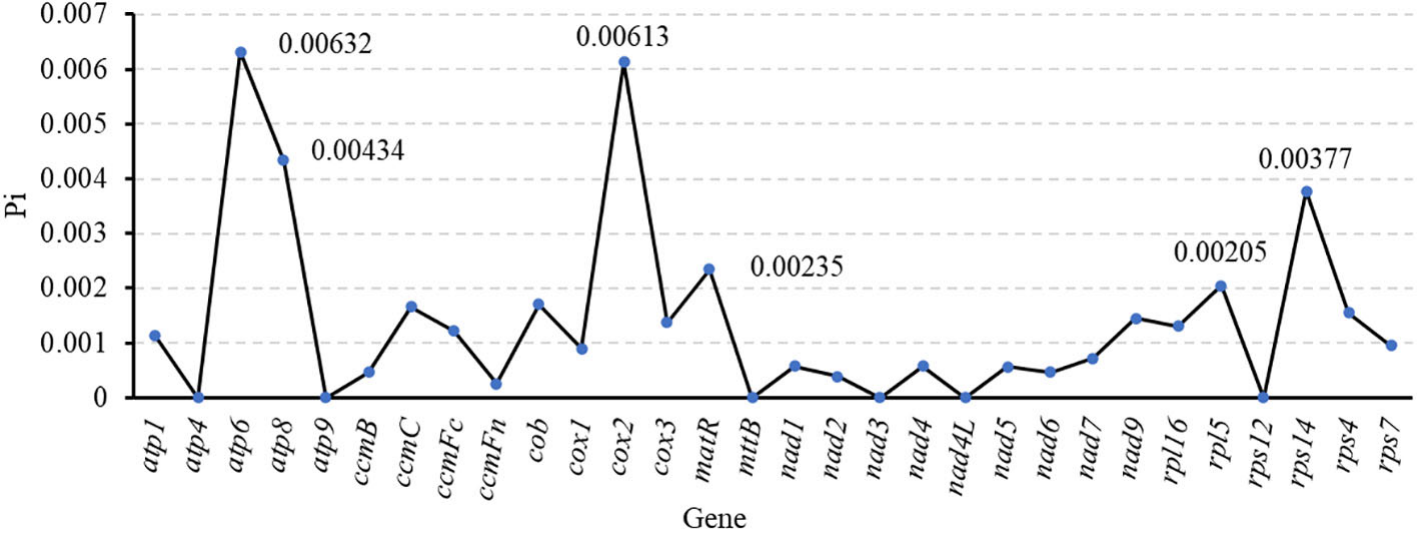
Nucleotide diversity of sseven *Medicago* species (30 shared genes).

In this study, the nucleotide diversity index (Pi) of 30 common genes in seven *Medicago* species was analyzed to reveal their genetic variation characteristics (**Figure 11**, **Supplementary Table S12**). Some genes showed high nucleotide diversity, for example, *atp6* (0.00632), *atp8* (0.00434), *cox2* (0.00613), *matR* (0.00235), *rpl5*(0.00205), *rps14*(0.00377), their Pi were greater than 0.002, these genes may have experienced relatively loose selection pressure or adaptive evolution. On the contrary, the Pi values of *atp4*, *atp9*, *mttB*, *nad3*, *nad4L* and *rps12* were zero, indicating that these genes were highly conserved in legumes. These results not only show the functional differentiation pattern of evolutionary conserved regions and variable regions in the mitochondrial genome of legumes, but also provide important clues for understanding the adaptive evolution mechanism of the genus. By comparing the nucleotide diversity of different genes, we can further explore the role of mitochondrial genome in species evolution and its potential biological significance.

### 3.8 Phylogenetic analysis

**Figure 12:**
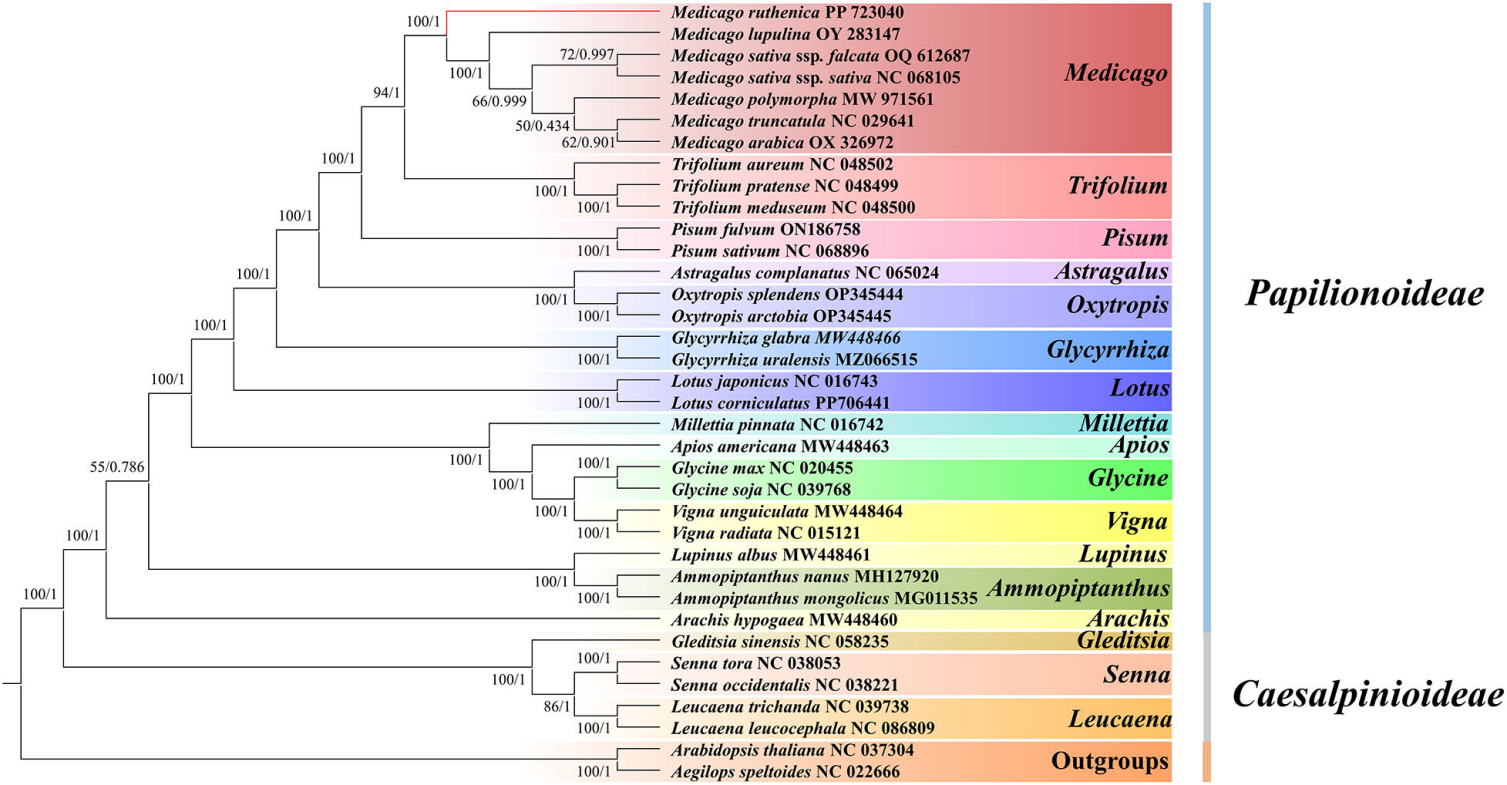
Evolutionary relationships inferred using 18 shared PCGs from the mitochondrial genomes of 30 Fabaceae species. Maximum Likelihood (ML) bootstrap support values and Bayesian posterior probabilities are provided for each node.

In this study, based on 18 mitochondrial common genes (*atp4*, *atp8*, *ccmC*, *cob*, *cox1*, *cox3*, *matR*, *nad2*, *nad3*, *nad4*, *nad5*, *nad6*, *nad7*, *nad9*, *rpl16*, *rps12*, *rps3* and *rps4*), the phylogenetic trees of 34 legume species were constructed to determine the evolutionary status of *M. truncatula*. *A.* sp*eltoides* and *A. thaliana* served as outgroups. Our results reveal identical topology in the phylogenetic trees constructed by RAxML (ML) and MrBayes (BI) analyses (**Figure 12**). Maximum Likelihood (ML) analysis showed that about 88.24% (30/34) of the nodes showed strong support (bootstrap value≥70%), of which 27 nodes (79.41%) reached full support (bootstrap value = 100%). Bayesian inference (BI) results further verify the topological reliability, and 91.18% of the nodes (31/34) have a posterior probability ≥ 0.95. In addition, the phylogenetic tree topology supports the differentiation of the 36 species in this study into ten branches.

The first clade is composed of *Medicago* and *Trifolium*. Seven species of *Medicago* (*M.ruthenica*, *M. truncatula*, *M. polymorpha*, *M. sativa* ssp*. falcata*, *M. sativa* ssp*. sativa*, *M. arabica* and *M. lupulina*) are clustered into a small clade, and three species of *Trifolium* (*T. aureum*, *T.meduseum* and *T.pratense*) are clustered into another small clade. The second largest clade consists of two species of *Pisum* (*P. fulvum* and *P. sativum*). The third branch is composed of Astragalus and Oxytropis. Among them, *A. complanatus* is a small branch, *Oxytropis* splendens and *O. arctobi* are clustered into another small branch. The fourth clade consists of two species of *Glycyrrhiza* (*G. uralensi* and *G.glabra*). The fifth branch consists of two species of *Lotus* (*L. corniculatu* and *L. japonicus*). The sixth branch was composed of *Milletti*, *Apios*, *Vigna* and *Glycine*, in which *M. pinnata* and *A. americana* were separated into a small branch, *V. radiata* and *V. unguiculata* were clustered into a small branch, and *G. uralensis* and *G. glabra* were clustered into another small branch. The seventh branch is composed of *Lupinus* and *Ammopiptanthus*, in which *L. albus* is a small branch alone, *A. mongolicus* and *A. nanus* are clustered into another small branch. The eighth branch is composed of *A. hypogaea* of *Arachis*. The ninth branch was composed of *Gleditsia*, *senna* and *Leucaena*, in which *G. sinensis* was separated into a small branch, *S. tora* and *S. occidentalis* were clustered into a small branch, and *L. trichanda* and *L. leucocephala* were clustered into another small branch. The tenth clade is the outgroup, which is composed of *A. thaliana* of *Brassicaceae* and *A.* sp*eltoides* of Brassicaceae.

### 3.9 Collinearity analysis

**Figure 13:**
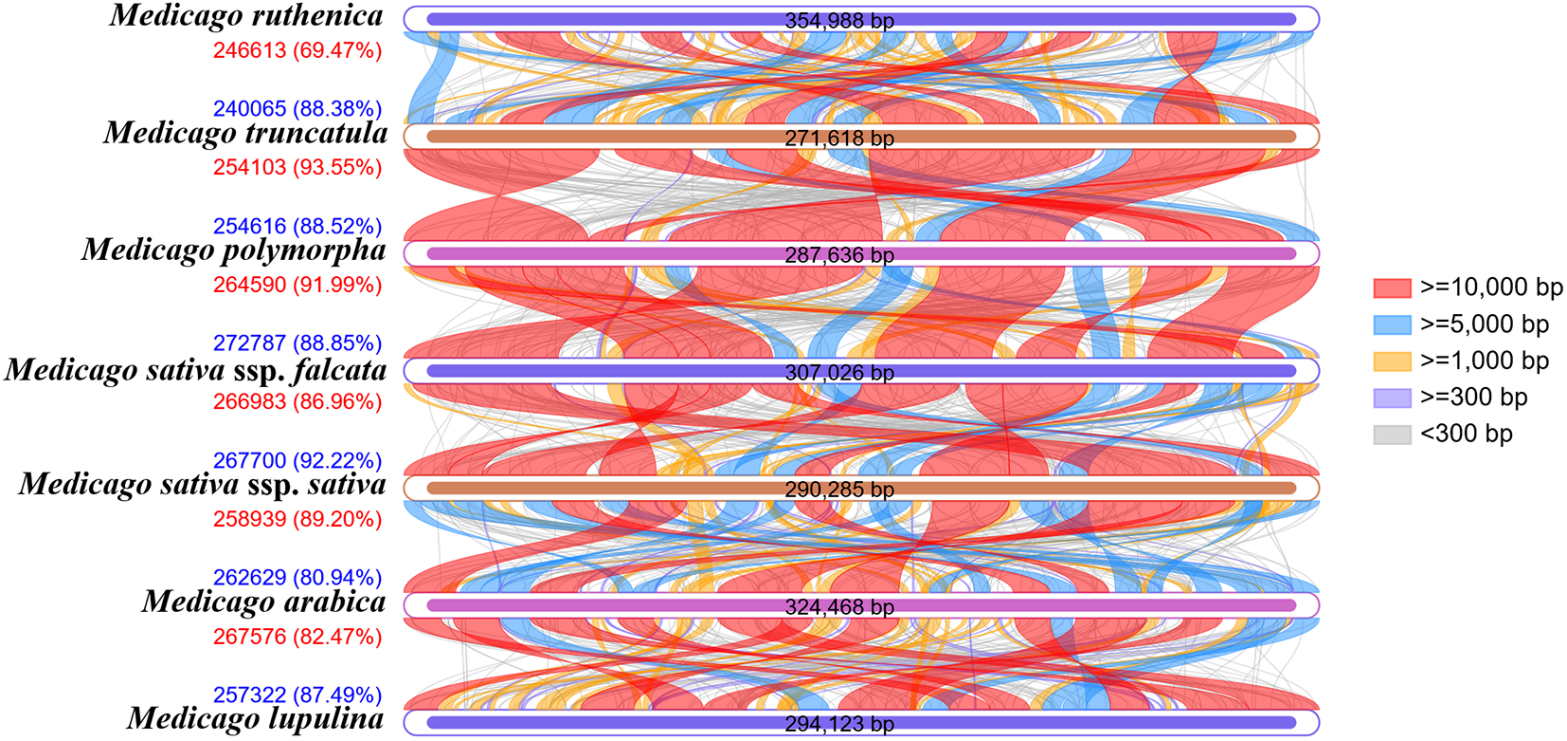
Collinearity analysis of seven *Medicago* species.

Collinearity analysis reveals the evolutionary associations between species by exploring the conservation of homologous gene sequences (**Figure 13**, **Supplementary Table S13**). In this study, the mitochondrial genomes of the seven *Medicago* species were compared pairwise. The length of homologous regions between *M. truncatula* and *M. polymorpha* was the largest, at 254,103 bp (93.55% of its mitochondrial genome) and 254,616 bp (88.52%), respectively, indicating that the two species had high similarity and conservation at the genomic level. Further analysis showed that the homologous regions of *M. polymorpha* and *M. sativa* ssp*. falcata* also accounted for 91.99% and 88.85%, respectively, also indicating high similarity. The homologous regions of *M. sativa* ssp*. falcata* and *M. sativa* ssp. *sativa* accounted for 86.96% and 92.22%, respectively. The homologous regions of *M. sativa* ssp. *sativa* and *M. arabica* accounted for 89.20% and 80.94%, respectively. The homologous regions of *M. arabica* and *M. lupulina* accounted for 82.47% and 87.49%, respectively. The coverage of homologous regions between *M. ruthenica* and *M. truncatula* was the lowest, 69.47% and 88.38%, respectively, suggesting that there were differences in genomic structure between them. In summary, *M. truncatula* is most closely related to *M. polymorpha*, whereas *M. ruthenica* is relatively distantly related to *M. arabica*. These results are consistent with the phylogenetic relationship of the seven *Medicago* species as inferred from the phylogenetic tree.

## 4 Discussion

Mitochondria, as essential semiautonomous organelles harboring their own genetic material in most eukaryotes, encode certain RNAs and polypeptides, and typically function in energy metabolism, development, and reproduction (Dyall et al., 2004; Kuzmin et al., 2005). Despite continuous advancements in sequencing technologies, plant mitochondrial genomes are increasingly variable and diverse. The size, structure, and gene content of mitochondrial genomes vary dramatically across angiosperms. The complexity of assembling plant mitochondrial genomes has led to a lag in the study of plant mitochondrial genomes (Knoop, 2013). In this study, we report the basic characteristics of the *M. ruthenica* mitochondrial genome for the first time. Our findings provide a valuable foundation for understanding the genomic function, inheritance, and evolutionary trajectory of *M. ruthenica*.

### 4.1 Mitochondrial genome analysis of *M. ruthenica*

We obtained a circular mitochondrial genome of 354,988 bp in length, which is larger than those of *M. truncatula* (271,618 bp), *M. polymorpha* (287,636 bp), *M. sativa* ssp*. falcata* (307,026 bp), *M. sativa* ssp*. sativa* (290,285 bp), *M. arabica* (324,468 bp) and *M. lupulina* (294,123 bp), but similar to those of *T. meduseum* (348,724 bp) and *O.* sp*lendens* (361,579 bp). The expansion of non-coding regions may have contributed to the evolution of larger and more complex plant mitochondrial genomes (Goremykin et al., 2012). A high guanine-cytosine (GC) content is common in plants (Smith and Keeling, 2015), reported GC contents of legume mitochondrial genomes range from 44.67% to 45.45%. Notably, the *M. ruthenica* mitogenome exhibits a GC content of 45.13%, which is falls within the GC content range (44-46%) of the related species of *Medicago*, including *M. truncatula* (45.39%), *M. polymorpha* (45.26%), *M. sativa* ssp*. falcata* (45.11%), *M. sativa* ssp*. sativa* (45.34%), *M. arabica* (44.92%) and *M. lupulina* (45.36%). Generally, plants species with a GC-biased mitochondrial genome also GC-rich in plastid genome (Smith and Keeling, 2015). GC base pairs with three hydrogen bonds were stronger than AT base pairs (two hydrogen bonds), resulting in greater stability of the *M. ruthenica* mitochondrial genome sequence. Higher GC content generally correlates with increased DNA stability (Chen and Skylaris, 2021). High GC content is often associated with functional advantages, such as stable gene expression and regulatory roles, which may be favored by natural selection (Muyle et al., 2011). Conversely, elevated GC content can lead to increased mutation and recombination rates, potentially enhancing genetic diversity and adaptability to changing or extreme environments (Kiktev et al., 2018).

Codon usage bias is of great significance in the mitochondrial genome. It not only affects the efficiency and accuracy of protein synthesis, but also has a close relationship with the adaptability and evolution of organisms (Jia and Higgs, 2008). This study systematically revealed the evolutionary characteristics of codon bias in the mitochondrial genomes of *M. ruthenica* and *M. truncatula*, *M. polymorpha*, *M. sativa* ssp*. falcata*, *M. sativa* ssp*. sativa, M. arabica* and *M. lupulina*. The results showed that the seven *Medicago* species exhibited significant codon usage conservation while maintaining the standard genetic coding framework. Among them, the types and quantities of preferred codons (RSCU > 1) of *M. truncatula*, *M. polymorpha*, *Medicago sativa* ssp*. falcata*, *M. sativa* ssp*. sativa* and *M. lupulina* were completely consistent. Notably the UGA terminator codon (Ter) of *M. ruthenica* indicated a preferential usage (RSCU = 1.29), while the UGA terminators of other species showed a non-preferential usage (RSCU ≤ 1). This difference may indicate the fine-tuning of the mitochondrial genome of *M. ruthenica* in the termination codon recognition mechanism, such as the regulation of tRNA abundance or the adaptive adjustment of translation termination efficiency. This species-specific feature may be related to its unique ecological adaptability, but the underlying molecular mechanism requires verification by tRNA expression profile and translation elongation kinetics experiments (Wu C. et al., 2019). We found that most PCGs in *M. ruthenica* exhibited a codon usage bias toward A/U-ending codons, which is consistent with the A/U preference observed in the chloroplast genome (Unseld et al., 1997). Monocotyledonous plants such as *Zea mays* typically show an enrichment of C/G-ending codons, whereas dicotyledonous plants such as *Taraxacum* species, have higher proportions of A/U-ending codons (Sharma et al., 2019; Tian et al., 2021; Fennoy and Bailey-serres, 1993). This A/U preference may reflect evolutionary adaptation, as A/U-rich codons dominate early genetic code evolution and could be shaped by amino acid functional spectra or gene expression optimization. However, natural selection and mutational pressures have also been recognized as significant drivers of codon usage bias (Parvathy et al., 2022; Yang et al., 2024).

This finding provides a novel entry point for the subsequent study of the association between mitochondrial genome evolution and species differentiation. In addition, only seven closely related species of *Medicago* are included as research samples, and the sample range is narrow. The limited number of samples may not fully and accurately reflect the overall pattern of mitochondrial genome codons in the entire *Medicago* species. Further studies are urgently needed to further expand the sample range, covering more *Medicago* species with different ecotypes and geographical distribution, as well as other plant groups with different genetic relationships with *Medicago*, and to carry out more extensive and in-depth comparative analysis, so as to fully verify the generalizability of the patterns identified herein and provide a more robust foundation for further understanding the theory of plant mitochondrial genome evolution.

### 4.2 Mitogenome repeats of *M. ruthenica*

Repeat sequences are critical factors in mitogenomic structural variation. The variations of mitogenome size and structure are thought to be caused by repeat-mediated duplication and recombination (Liu et al., 2024). We identified 148 dispersed repeats in the mitochondrial genome, with no inverted or complementary repeats, a pattern observed in the *M. ruthenica* chloroplast genome. Mitogenome copy numbers may predict plant evolutionary rates, the lower copy numbers may provide fewer templates, homologous recombination may act efficiently with a higher copy number (Zwonitzer et al., 2024). These 148 repeats belong to the imperfect repeats, are prevalent in mitogenomes, and are characterized by short, evolutionarily dynamic sequences (Fontecilla-Camps, 2023). Short insertional repeats are abundant in mitogenomes owing to their flexible nature and susceptibility to breaks, gene recombination, and conversion during plant evolution (Liu et al., 2024). Whereas the higher abundance of long repeats indicates that the mitochondrial genome may undergo more recombination events (Wang et al., 2025). In contrast, a lower abundance of long repeats or a higher abundance of short repeats in *M. ruthenica* suggests that its mitochondrial genome may undergo fewer recombination events during evolution. These short repeats are particularly frequent and large in vascular plants, potentially signaling adaptive strategies for their survival and evolution (Shakyawar et al., 2009). The presence or absence of inverted repeats significantly affects mitochondrial genome stability, replication, and evolution. Angiosperms such as *Silene noctiflora* exhibit enhanced genomic stability owing to the lack of inverted repeats (Kanazawa et al., 1998). Conversely, inverted repeats in other species facilitate complex rearrangements and influence replication mechanisms (Wynn and Christensen, 2019). Recent reports have indicated that the yellow-flowered *M. ruthenica* harbors large inverted repeats in its chloroplast genome, suggesting evolutionary novelty (Wu et al., 2015). Similarly, *M. minima*, despite being an ancestral member of the IRLC, lost a 9 kb inverted repeat, which may explain differences in reproductive modes and environmental adaptation during evolution (Choi et al., 2020). Understanding these structural elements is crucial for unraveling the evolutionary complexity of mitogenomes across plant species.

The SSRs contribute to genomic evolution and diversity through rapid evolutionary changes associated with high mutation rates (Čechová et al., 2018). SSRs are classified as mono-, di-, tri-, tetra-, penta-, or hexanucleotide repeats. In this study, 72 SSRs were identified in the *M. ruthenica* mitogenome, accounting for 1.10% of its mitochondrial genome, a proportion relatively close to that of *M. truncatula* (2.41%) (Wen et al., 2024). Dinucleotide (33.33%) and tetranucleotide (29.17%) repeats predominated, with A/T bases constituting 98.61% of all SSRs. Mononucleotide and dinucleotide repeats are common in legume species, including *Ormosia boluoensis*, *A. mongolicus*, and *G. sinensis* (Li et al., 2018; Kumar et al., 2020; Liu et al., 2021; Xie et al., 2021), whereas tetranucleotide and hexanucleotide repeats are rare in plant mitogenomes, with the exception of *Trigonella foenum-graecum* (Yu et al., 2018; Yang et al., 2021). A/T- or TA/AT-rich repeats are also prevalent in legumes, such as *M. truncatula*, *T. foenum-graecum*, and *G. sinensis* (Li et al., 2018; Kumar et al., 2020; Liu et al., 2021). Most SSRs were distributed in the IGS regions, a pattern observed in other plants, such as *E. sibiricus* and *M. polymorpha* (Choi et al., 2020; Zhang et al., 2023). SSRs have potential as molecular phylogenetic markers because of their variability and distribution; however, their application in phylogenetic studies is challenged by the nonlinear dynamics in mitochondrial genomes (Choi et al., 2020).

### 4.3 RNA editing events and number of *M. ruthenica*

Mitochondrial rRNAs and tRNAs play crucial roles in cellular stress responses and resistance mechanisms as mitochondrial DNA acts as a stress sentinel that can activate nuclear DNA damage and repair pathways, while rRNAs and tRNAs are involved in post-transcriptional processing (Zhao et al., 2016). The *M. ruthenica* mitogenome contains three rRNAs genes (*rrn18*, *rrn26*, and *rrn5*), a feature shared with most *Medicago* species; however, its 18 tRNA genes exceed the numbers reported in Zhongmu No.1, Zhongmu No.4, *M. minima*, and *M. truncatula* (Nishikawa et al., 2005; Choi et al., 2020; Kumar et al., 2020; Liu et al., 2021). The relationship between tRNA gene numbers in mitochondrial genomes and resistance mechanisms is complex, as approximately 25% of tRNAs exist in organellar genomes (Wu Z. et al., 2019). These tRNAs may function as regulators of stress responses, by generating stress-induced tRNA-derived fragments (Matsui et al., 2013), which could enhance *M. ruthenica* tolerance to abiotic stress.

The post-transcriptional modification of RNA molecules, known as mitochondrial RNA editing, alters their nucleotide sequences and plays a vital role in regulating plant stress resistance. Our results indicate that C-to-U editing occurs in the *M. ruthenica* mitochondrial genome, a phenomenon conserved across plant species such as *A. thaliana*, *Triticum aestivum* and *Poa pratensis* (Sablok et al., 2017; Hu et al., 2021; Ai et al., 2024). Editing is essential for accurate mitochondrial protein coding, and is influenced by selective pressures. C-to-U editing modifies codons to increase protein hydrophobicity; these hydrophobic proteins are typically involved in stress responses and signaling pathways (Gualberto et al., 1991; Giegé and Brennicke, 1999). RNA editing rates can be modulated by environmental stresses; for example, C-to-U editing decreases under heat or cold stress in *Arabidopsis* but increases under alkaline stress in *Oryza sativa* (Gosslau et al., 2001). Understanding these RNA editing mechanisms could facilitate the development of crops with enhanced stress tolerance.

### 4.4 Ka/Ks and Pi analysis of *M. ruthenica*

The Ka/Ks value, the ratio of non-synonymous to synonymous substitutions, is often used to evaluate the evolutionary rate of genes under selection pressure (Park et al., 2018). When Ka/Ks>1, it indicates that the gene is under purified selection, suggesting that the gene has undergone adaptive changes in evolution, which may be related to its functional adjustment under specific environmental pressures (Yang and Bielawski, 2000). In this study, the dynamic role of natural selection pressure in the evolution of extranuclear genomes was revealed by analyzing the Ka/Ks values of 26 common genes in the mitochondrial genomes of 34 legume species. The Ka/Ks values of most genes (such as *cox1*, *cob*, *nad7* and *nad4L*) were significantly lower than 0.3, indicating that these genes were subjected to strong purification selection pressure. These genes participate in the electron transport, proton pump and ATP synthesis of the mitochondrial electron transport chain by encoding the key subunits of the respiratory chain complex. Their highly conserved sequence features are likely essential for maintaining the core function of mitochondrial energy metabolism (Sloan et al., 2012). In this study, some genes showed significant positive selection signals (Ka/Ks > 1) in specific lineages, and the functional optimization of these genes may directly enhance the adaptability of plants to adversity (Begum and Mondal, 2021). The Ka/Ks values of some genes were close to the neutral threshold (0.976155-1.09295), suggesting that they may be in the transition stage from purified selection to positive selection (Yang et al., 2023). For example, the *ccmFc* and cox*3* genes of *P. fulvum*, the *nad4* gene of *G. max*, the *nad9* gene of *A. hypogaea* and the *ccmB* gene of *G. sinensis* all showed such characteristics. This dynamic change provides a key clue for the study of selection pressure conversion, which is helpful to understand the evolution of gene function under different environmental pressures. Plants species with high copy numbers result in relatively slow “normal” substitution rates of mitochondrial genomes in most plant species. Lower copy numbers lead to various evolutionary rates, with some species experiencing rate acceleration having relatively lower copy numbers, while others with low copy numbers maintain low substitution rates without experiencing rate acceleration (Zwonitzer et al., 2024; Wang et al., 2024b).

Nucleotide diversity index (Pi) is one of the important means to measure the genetic diversity of species. It reflects the degree of genetic variation within the population by calculating the average nucleotide difference between each pair of sequences in a specific gene region (Konopiński, 2022). The high Pi usually means that the species has rich genetic diversity, which is of great significance for their adaptation to environmental changes, resistance to diseases and long-term evolution (Massa et al., 2024). In botanical studies, the PCGs in the mitochondrial genome are considered to be ideal for assessing genetic diversity due to their conserved and easy analyze characteristics (Wang et al., 2023). Among the seven *Medicago* species studied, some genes showed relatively high Pi values (ranging from 0.00205 to 0.00613), which may reveal the adaptive fine-tuning or relaxation of environmental selective pressure experienced by these genes during evolution (Mousavi-Derazmahalleh et al., 2018). Specifically, oxidoreductase-related genes (*cox2*) and ATP synthase-related genes (*atp6*) showed significant genetic diversity. Among them, the Pi value of *cox2* gene is as high as 0.00632, and the Pi value of *atp6* gene is 0.008724. This genetic variation may enable *M. ruthenica* to more effectively regulate its energy metabolism processes in different environments to adapt to specific ecological conditions. In addition, the ribosomal protein gene *rps14* also showed high genetic diversity (Pi = 0.00377). As the key structure of intracellular protein synthesis, ribosome function depends on the involvement of various ribosomal proteins. The ribosomal protein encoded by *rps14* gene plays an important role in the ribosome assembly. The variation of *rps14* gene may optimize the rate of ribosome assembly, so that *M. ruthenica* can respond more flexibly to changes in translation needs (Figueroa et al., 1999). This mechanism may help plants quickly adjust the rate of protein synthesis to maintain normal physiological functions in the face of environmental stress. Taken together, the high genetic diversity of these genes may reflect the adaptive adjustment strategies of *Medicago* plants in specific habitats. Therefore, in-depth exploration of the functions of these genes and their interactions with the environment can not only help to reveal the evolutionary history of *Medicago* plants, but also provide key scientific support for future conservation strategies and breeding practices.

### 4.5 Phylogenetic relationship of *M. ruthenica*

Since the establishment of *Medicago*, this genus has become an important research direction in botany. To date, the reported taxonomic studies for *M. ruthenica* have employed methods such as SSR/ISSR analysis, ITS2 sequence analysis at the molecular level (Takezawa and Minami, 2004), flavonoid compound type analysis at the chemical level, pollen morphology or karyotype analysis at the cellular level (Rehman et al., 2022), and chloroplast genome analysis at the organelle level (Goremykin et al., 2012; Wu et al., 2015; Wen et al., 2024). Nonetheless, there is an absence of complete mitochondrial genome data for this species needed for phylogenetic studies. In this study, based on 18 mitochondrial common genes, *A.* sp*eltoides* and *A. thaliana* were selected as outgroups to construct a phylogenetic tree of 34 legume species, and the phylogenetic status of *M. ruthenica* was clarified. In addition, Phylogenetic analysis further revealed that *M. ruthenica* and *M. truncatula*, *M. polymorpha*, *M. sativa* ssp*. falcata*, *M. sativa* ssp*. sativa*, *M. arabica and M. lupulina* were clustered into the same branch in the phylogenetic tree, which further supported the taxonomic placement of *M. ruthenica* within *Medicago*. *M. ruthenica* was placed within the *Medicago* clade, but formed an independent branch. Phylogenetic analysis strongly supports a shared ancestry with other *Medicago* species, indicating *M. ruthenica* represents a distinct species within the genus (Xu et al., 2021). The branch lengths in phylogenetic trees reflect evolutionary divergence time. Here, *M. ruthenica* exhibited significantly longer branch lengths than did the other six congeneric species, suggesting either prolonged evolutionary divergence or later speciation compared with that of its relatives. The evolutionary history of *Medicago* likely involves survival challenges due to severe environmental changes. Chloroplast genome analysis of 61 *M. ruthenica* accessions revealed two major clades that diverged during the Last Glacial Period (Yan et al., 2021). Organelle-based molecular analyses can effectively explore genetic relationships and evolutionary processes in *M. ruthenica*, despite the limitations of high substitution rates and potential phylogenetic signal biases (Li et al., 2013).

This study expands the mitochondrial genome resources and provides novel insights into *M. ruthenica* classification. In conclusion, the results of this study not only reinforce certain theoretical foundations of traditional taxonomy, but also highlight the important value of mitochondrial genome in plant phylogenetic research. In addition, both maximum likelihood and Bayesian inference analyses were employed in this study, and the results showed high node support values, which indicated that mitochondrial genes had high reliability in analyzing the evolutionary relationship of legume species. At the same time, the topological structure of the phylogenetic tree constructed herein is consistent with previously reported legume phylogenies, which further verifies the credibility of the research results. Although the phylogenetic tree constructed based on mitochondrial genes in this study provides an important reference for the evolutionary relationship of legume species, there are still some limitations. At present, reports on the mitochondrial genome of legume plants are relatively scarce. Therefore, future studies should therefore aim to expand the sample size, which will help to more comprehensively and deeply analyze the evolutionary history of legume species and their complex evolutionary mechanisms.

## 5 Conclusions

The *M. ruthenica* mitochondrial genome spans 354,988 bp, approximately 2.80-fold larger than its chloroplast counterpart (126,939 bp), with a GC content of 45.13%. It contains 34 PCGs 18 tRNA genes, and 3 rRNA genes (*rrn18*, *rrn26*, *rrn5*). The mitogenome harbors 72 SSRs, with the dinucleotide (24) and tetranucleotide (21) repeats being the most abundant. The RSCU value of the codon UUA encoding Leu was the largest (1.55), the number of codon UUU encoding Phe was the largest (257-341), and the number of codon UAG encoding Ter was the least (4-5). Ka/Ks value analysis showed that genes such as *atp8*, *atp4*, *rps4*, *nad4*, *ccmB*, *ccmFc* and *nad9* showed significant positive selection in multiple legumes (Ka/Ks > 1). Furthermore, phylogenetic tree revealed *M.ruthenica* belongs to the genus *Medicago* and is a separate branch. These results provide an valuable model for studying *M. ruthenica* evolution and offer critical referenc for mitochondria-associated traits in *M. ruthenica* breeding.

## Data Availability

The datasets presented in this study can be found in online repositories. The names of the repository/repositories and accession number(s) can be found in the article/**Supplementary Material**.
